# ToF‐SIMS analysis of a polymer microarray composed of poly(meth)acrylates with C_6_ derivative pendant groups

**DOI:** 10.1002/sia.5959

**Published:** 2016-02-19

**Authors:** Andrew L. Hook, David J. Scurr

**Affiliations:** ^1^ Laboratory of Biophysics and Surface AnalysisUniversity of NottinghamNottinghamNG7 2RDUK

**Keywords:** surface analysis, polymer microarray, multivariate analysis, high‐throughput surface characterisation

## Abstract

Surface analysis plays a key role in understanding the function of materials, particularly in biological environments. Time‐of‐flight secondary ion mass spectrometry (ToF‐SIMS) provides highly surface sensitive chemical information that can readily be acquired over large areas and has, thus, become an important surface analysis tool. However, the information‐rich nature of ToF‐SIMS complicates the interpretation and comparison of spectra, particularly in cases where multicomponent samples are being assessed. In this study, a method is presented to assess the chemical variance across 16 poly(meth)acrylates. Materials are selected to contain C_6_ pendant groups, and ten replicates of each are printed as a polymer microarray. SIMS spectra are acquired for each material with the most intense and unique ions assessed for each material to identify the predominant and distinctive fragmentation pathways within the materials studied. Differentiating acrylate/methacrylate pairs is readily achieved using secondary ions derived from both the polymer backbone and pendant groups. Principal component analysis (PCA) is performed on the SIMS spectra of the 16 polymers, whereby the resulting principal components are able to distinguish phenyl from benzyl groups, mono‐functional from multi‐functional monomers and acrylates from methacrylates. The principal components are applied to copolymer series to assess the predictive capabilities of the PCA. Beyond being able to predict the copolymer ratio, in some cases, the SIMS analysis is able to provide insight into the molecular sequence of a copolymer. The insight gained in this study will be beneficial for developing structure–function relationships based upon ToF‐SIMS data of polymer libraries. © 2016 The Authors Surface and Interface Analysis Published by John Wiley & Sons Ltd.

## Introduction

Surface analysis plays a key role in the development of materials as it is the surface of a material that will interact with its surrounding environment, thereby determining its function.^1^, ^2^, ^3^, ^4^, ^5^ As a material's surface can differ from the bulk,^6^ it is the surface properties rather than bulk composition that should be utilised to elucidate structure–function relationships used for further material optimisation for applications involving interfacial contact of the material with its surroundings.^7^, ^8^, ^9^, ^10^ This is particularly relevant when large libraries of materials or material gradients are being assessed as these systems allow the response of a certain environment to large groups or populations of materials to be assessed, and hence provide a robust insight into underlying interactions.^11^, ^12^, ^13^ The polymer microarray format has become a key enabling tool for materials discovery and development,^14^, ^15^, ^16^, ^17^, ^18^, ^19^, ^20^ whereby hundreds to thousands of unique polymers are printed onto a single glass slide allowing for parallel screening. Further to the identification of novel materials, the large number of biological–material interactions that can be assessed using high‐throughput screening methodologies can be used to provide new insight into structure–function relationships. For example, in a recent study, a correlation was observed between bacterial attachment to polyacrylates with a composite parameter derived from molecular descriptors associated with molecular rigidity and hydrophobicity. This was made possible by the large number of bacterial–material interactions that were assessed using the polymer microarray.^13^

In order to achieve surface analysis of high‐throughput systems the method must be automated such that hundreds to thousands of measurements can be acquired without generating a bottle‐neck within the high‐throughput materials development cycle. Automated surface analysis of large sample sets^21^ has been achieved for water contact angle measurements,^22^, ^23^ X‐ray photoelectron spectroscopy,^4^, ^11^, ^12^, ^24^ atomic force microscopy,^25^ surface plasmon resonance^26^, ^27^, ^28^ and time‐of‐flight secondary ion mass spectrometry (ToF‐SIMS).^29^, ^30^, ^31^, ^32^ ToF‐SIMS has been widely used for studying polymeric systems,^33^, ^34^, ^35^, ^36^, ^37^, ^38^, ^39^, ^40^, ^41^, ^42^, ^43^ and when coupled with multivariate analysis^44^, ^45^, ^46^ has proven to be particularly useful for establishing correlations between the surface chemistries of a library of materials with various properties such as water contact angle and cell attachment.^17^, ^44^, ^47^, ^48^, ^49^, ^50^, ^51^, ^52^ Whilst these correlations eloquently demonstrate a relationship between surface chemistry and the interfacial performance of the materials, the models themselves are difficult to interpret as they typically feature an abundance of small ion fragments that are derived from multiple, if not all, materials present in a study and are thus difficult to assign to specific chemical functionalities or properties.

In the present study, a method is presented for the analysis of the chemical variance across a polymer library. A total of 16 different (meth)acrylate monomers containing pendant groups derived from six carbons were selected as an example set for analysis. The ToF‐SIMS spectra were acquired for each material on a microarray, and the most intense and unique secondary ions were identified and used to interpret the variance within the SIMS spectra as assessed using principal component analysis (PCA).

## Methods

### Polymer microarray formation

Polymer microarrays were formed as previously described^53^, ^54^ using an AD3200 dispensing workstation (Biodot, Irvine, CA, USA). Epoxy‐functionalized glass slides (Molecular Devices, Sunnyvale, CA, USA) were dip coated in 4% (w/v) poly(hydroxy ethylmethacrylate) (pHEMA) (Sigma‐Aldrich, St. Louis, MO, USA, cell culture tested) in ethanol at a withdrawal rate of approximately 30 mm/s. The slides were held horizontally for 1 min to allow solvent evaporation before placing in a drying rack for 3 days. Polymerisation solution composed of 75% (v/v) monomer (Sigma‐Aldrich) in dimethylformamide with 1% (w/v) photoinitiator 2,2‐dimethoxy‐2‐phenylacetophenone was printed onto the pHEMA coated slides using 946PM6B pins (Arrayit, Sunnyvale, CA, USA) at O_2_ < 2000 ppm, 25 °C and 40% humidity. After printing each material, slides were irradiated with a long wave UV source for 30 s. Slides were irradiated for a further 10 mins once all materials had been printed. The UV likely induced cross‐linking, which may limit comparison of the spectra presented in this manuscript with polymers prepared using a different method. Once array formation was complete, the slides were vacuum extracted at <50 mTorr for 5 days.

### Optical microscopy

Phase contrast images were acquired on an Olympus IX51 microscope and a Smart Imaging System (Imstar SA, Paris, France) with a 4× objective lens. Image mosaics were reconstructed using Pathfinder^TM^ software (Pathfinder Development, Chicago, IL, USA).

### Time‐of‐flight secondary ion mass spectrometry

Time‐of‐flight secondary ion mass spectrometry measurements were conducted using a ToF‐SIMS IV (IONTOF GmbH, Münster, Germany) instrument operated using a 25 keV Bi_3_^+^ primary ion source exhibiting a pulsed target current of >0.3 pA. Samples were scanned at a pixel density of 512 pixels per mm, with eight shots per pixel over a given area. An ion dose of 2.45 × 10^11^ ions per cm^2^ was applied to each sample area ensuring that static conditions were maintained throughout. Both positive and negative secondary ion spectra were collected (mass resolution of >7000 at *m*/*z* = 29), over an acquisition period of 30 scans (the data from which were added together). Owing to the non‐conductive nature of the samples, charge compensation was applied in the form of a low energy (20 eV) electron floodgun. Patch areas of 0.5 × 0.5 mm were acquired at a resolution of 256 × 256 pixels by rastering the primary ion beam over the patch using a ‘random raster’ path sequence. Patch areas were sequentially acquired over the entire microarray using programmed stage movements through the macro‐raster stage function. The patch areas were combined into a mosaic image, allowing all patches to be processed together. Ions associated with the pHEMA background, such as C_2_H_5_O^+^,^29^ were used to extract the regions associated with the printed polymers, which were used to calibrate the spectra and produce a peak list using a peak search tool (SurfaceLab 6, IONTOF), minimum counts set to 100, maximum background set to 0.8. To ensure the peak search tool had successfully identified peaks, any ions of interest were visually inspected. Regions associated with each polymer spot were then extracted and recalibrated, and the peak list was applied to produce an individual spectrum for each polymer. In total, 436 positive and 855 negative ion peaks were identified.

### Peak assignment

Assignment of peaks was assisted by a custom built Visual Basic for Applications algorithm (PeakAssigner v2.5), which sequentially built chemical structures from a set number of elements and identified structures matching the mass of a particular ion within an allowed deviation. Unpaired valence electrons were used to assign bonds between atoms in order to identify stable chemical structures. For a molecule containing *n* atoms, the minimum number of bonds was limited to *n*‐1. No more than three bonds were allowed between two atoms. The total number of allowed unpaired valence electrons and the degree of saturation within a chemical structure were adjustable and were typically set to 5 and 10, respectively. This was rapidly achieved for all 727 peaks with control over the type and frequency of elements and the maximum degree of unsaturation for each mass. All peak assignments were below 75 ppm deviation. The series of possible assignments were compiled into a list for each mass allowing easy comparison between different but related masses (e.g. masses with high loadings for the same principal component) to assist with assignment and to highlight fragmentation trends. The Visual Basic for Applications algorithm and accompanying Excel spreadsheets PeakAssigner (v2.6) are available in the supporting information and are described in detail in the supporting information. The software along with exported SIMS spectra from the 16 polymers used in this study are available on the NESAC/BIO website.^55^ A built in tutorial is included within the Excel speadsheet.

### Principal component analysis

Both positive and negative peak intensities were dead time corrected^56^ and subsequently normalised to their respective total secondary ion counts to remove the influence of primary ion beam fluctuation. The positive and negative ion intensity data were split into a training set featuring seven sample replicates and a test set featuring the remaining three sample replicates. Each data matrix was square root mean scaled and mean‐centred to the mean of the training set prior to analysis.^57^, ^58^, ^59^ The same pre‐processing was used when principal component (PC) scores were determined for the copolymer series. PCA was carried out using PLS Toolbox 5.2 software (Eigenvector, Wenatchee, WA, USA), an add‐on to MATLAB R2009b. The number of principal components was determined from an eigenvalue plot using a scree test and considering the principal components that explained 95% of the variance.^60^

## Results

**Figure 1:**
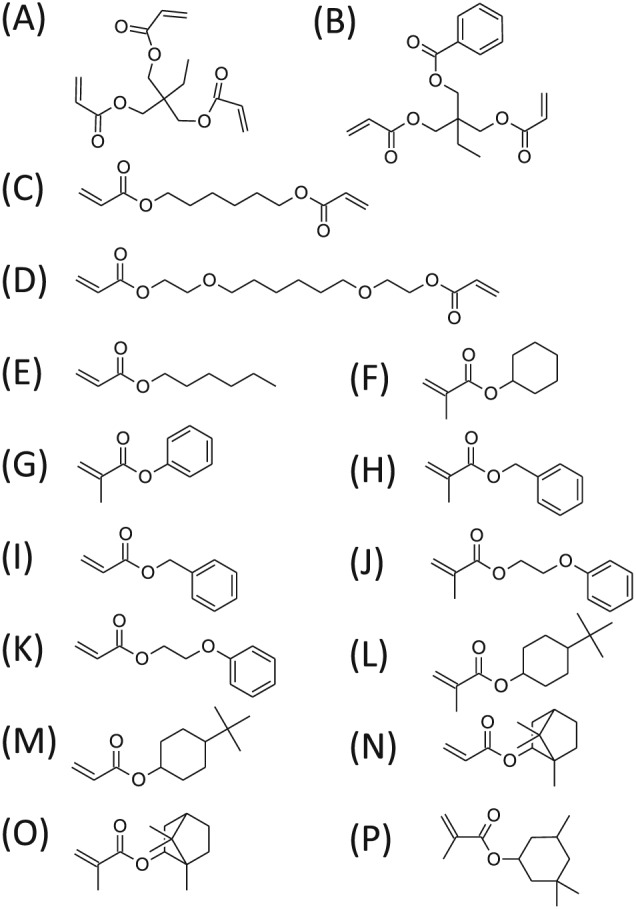
Chemical structures of the 16 monomers used to produce the polymers analysed within this study.

**Figure 2:**
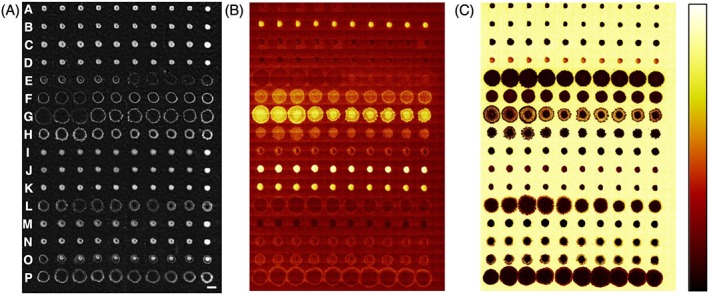
Assessment of the polymer microarray produced. Samples are arranged from top to bottom as produced from monomers A to P as indicated in (A). Replicate spots are across each row. (A) Mosaic of phase contrast images of the polymer microarray, scale bar = 500 µm. (B and C) ToF‐SIMS ion images from the microarray area corresponding to (B) C_6_H_5_^+^ and (C) C_2_H_5_O^+^. Images shown at same scale as (A). Intensity scale for (B) and (C) shown to the right of (C).

To create an example set of materials for ToF‐SIMS and multivariate analysis, a selection of 16 acrylate and methacrylate monomers (Fig. 1) that all contained pendant groups with moieties based upon six carbons (such as hexane, cyclohexane and benzene) were selected. The materials were printed as a polymer microarray with ten repeat spots per monomer. To assess the print quality of the array,^6^ phase contrast images (Fig. 2(A)) along with ToF‐SIMS ion images of the C_6_H_5_^+^ ion associated with the printed polymers and the C_2_H_5_O^+^ ion associated with the pHEMA background were produced (Fig. 2(B and C)). All 160 polymer spots were successfully printed and were distinct from other spots. A region of interest (Fig. SI1) was assigned for each polymer spot to obtain individual SIMS spectra, shown in Fig. SI2.

### Assessment of high intensity ions

**Figure 3:**
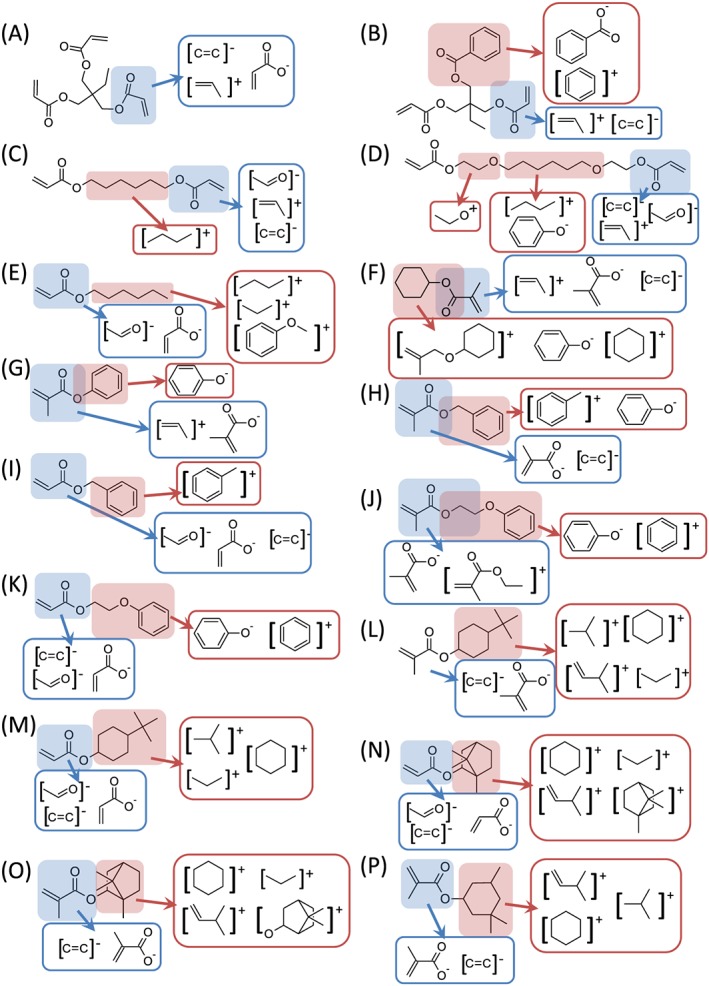
Chemical structures of the 16 monomers (A–P) along with the highest intensity ions observed for each material. The ions are grouped as originating from either the (meth)acrylate backbone (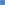) or the pendant group (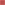).

**Figure 4:**
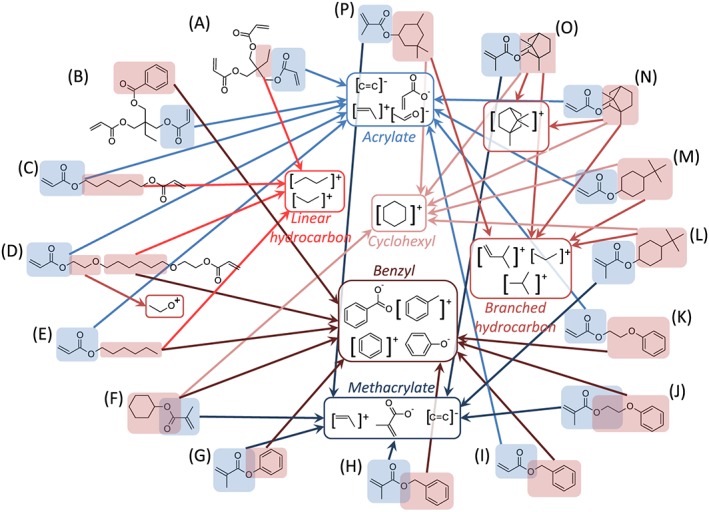
Summary of common ions derived from the 16 materials (A–P). The ions are grouped as originating from either the (meth)acrylate backbone (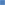) or the pendant group (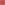).

Initially, the highest intensity secondary ions derived from each material were identified, listed in Table SI1. These ions were classified based upon whether they originated from the (meth)acrylate backbone or from the pendant groups (Fig. 3). A summary of the ions common to the 16 materials is provided in Fig. 4.

A high frequency of the most intense ions for a material was found to correspond to the stoichiometry of the respective pendant groups derived from cleavage of the C—O bond at the ester group (Fig. 3). Examples include the C_6_H_5_O^−^ ion from polyG and polyH, the C_6_H_11_^+^ ion from polyF and the C_9_H_13_O^+^ ion from polyN and polyO.

A number of characteristic ions for the methacrylate or the acrylate groups were observed (Fig. 4), most notably, the C_4_H_5_O_2_^−^ ion was observed for methacrylates whilst the C_3_H_3_O_2_^−^ ion was observed for acrylates, consistent with previous studies.^40^, ^43^ Generally, a higher intensity for the C_4_H_5_O_2_^−^ ion was observed for methacrylates compared with the intensity observed for the C_3_H_3_O_2_^−^ ion for acrylates; the C_4_H_5_O_2_^−^ or C_4_H_5_O^+^ ion was the first or second most intense ion for six of the eight methacrylates used in this study, whilst for the acrylates, the C_3_H_3_O_2_^−^ or C_3_H_3_O^+^ ions did not feature as the second most intense ion for any of the monoacrylates and was as low as the seventh most intense ion (Table SI1).

The spectra of the various polymers were assessed for the presence of C_6_ ions amongst the most intense ions for each material. C_6_ fragments were more prevalent for cyclic moieties as opposed to linear moieties, likely because the fragmentation of a cyclic structure first requires linearization, whereas linear structures can be shortened by breaking a single bond only. C_6_H_5_ or C_7_H_5_ (positive and negative) ions were readily observed in the SIMS spectra with and without oxygen for polymers that contained benzene (monomers B, G, H, I, J and K, Fig. 3). The number of carbons in the ions generated depended upon whether the benzyl group was directly bound to an oxygen atom or not, consistent with C—O acting as a cleavage point for ion generation.^39^, ^41^ For polymers of monomer F (polyF), which contained a cyclohexyl pendant group, a number of ions containing the cyclohexyl moiety were observed amongst the highest intensity ions within its spectrum, including C_6_H_11_^+^ and C_9_H_11_O_2_^+^. This observation is consistent with the structure of cyclic moieties being preserved during SIMS ion fragmentation. A number of the most intense ions observed for polyF were unsaturated, such as C_6_H_5_O^−^ and C_7_H_5_O^+^, despite there being no benzene groups present on the polymer. This suggests that the SIMS ion fragmentation process can induce extra degrees of unsaturation within the ions produced. It is therefore noted that post‐fragmentation changes in an ion can result in a spectrum that may seem to misrepresent a particular chemical species, particularly where stable ions such as benzene can be formed.^36^, ^39^, ^42^

The most intense ion observed for polymers that contained the *tert*‐butyl cyclohexyl moiety (monomers L and M) was C_4_H_9_^+^, likely originating from the *tert*‐butyl group.^48^ Additionally, a high intensity for the C_6_H_11_^+^ ion, derived from the cyclohexyl group, was also observed. Unlike polyF, there were no benzyl containing ions within the most intense ions observed for polyL and polyM. It is possible that the fragmentation pathway for the pendant group on these polymers caused the cyclohexyl group to linearise. This is evidenced by the high intensity for the C_5_H_9_^+^ ion that likely results from the loss of a methylene (CH_2_) group from the C_6_H_11_^+^ ion. This would be more easily achieved on a linear hydrocarbon fragment rather than a cyclic fragment. An ion representing both the cyclohexyl and *tert*‐butyl groups together was not observed amongst the most intense ions for either polyL or polyM, suggesting that this relatively large moiety is susceptible to fragmentation.

The most intense ion observed in the SIMS spectrum of polyN was C_6_H_9_^+^, which is thought to originate from the cyclohexyl ring in the isobornyl group. This ion was also predominant in the spectrum of polyO, the methacrylate counterpart of polyN. In total, four chemical bonds needed to be broken in order for this ion to be produced, suggesting that the removal of branched methyl or ethyl groups readily occurred within the SIMS fragmentation process. The reduced number of hydrogens on this ion, compared with the C_6_H_11_^+^ ion observed for cyclohexyl containing polymers of monomers L, M and F, may be caused by the removal of the branched species, suggesting that a higher intensity of ions with a H:C ratio below 2 may indicate a branched hydrocarbon. However, the C_6_H_11_^+^ ion was the most intense C_6_ ion observed for polyP, which originated from the trimethyl cyclohexyl group of polyP after the removal of three methyl groups. An ion representing the complete removal of the isobornyl group (C_10_H_17_^+^) was observed amongst the most intense ions for polyN.

The inclusion of multi‐functional monomers unsurprisingly caused an increase in the intensity of ions originating for the acrylate group, such as for polyA whose SIMS spectrum was dominated by the C_3_H_3_O_2_^−^, C_3_H_3_O^+^, and C_2_H^−^ ions that originate from the acrylate group.

High numbers of small carbon fragments were observed for polymers of monomers C, D and E that contained linear C_6_ groups. For example, the SIMS spectrum for polyE was dominated by the C_3_H_7_^+^ ion, which was more than twice as intense as any other. The ion likely originated from the hexane pendant group on this monomer. A C_7_H_5_O^+^ ion was observed for this material, which is proposed to originate from the hexane pendant group and a fragmentation event within the ester group. Benzene like fragments also featured within the SIMS spectra of polyC and polyD, such as C_6_H_5_^+^ and C_6_H_5_O^−^, respectively. This result further demonstrates the tendency for SIMS to preferentially generate stable ions such as benzene. No saturated C_6_ ions were observed within the most intense ions for any of the polymers with linear C_6_ pendant groups.

### Assessment of unique ions

**Figure 5:**
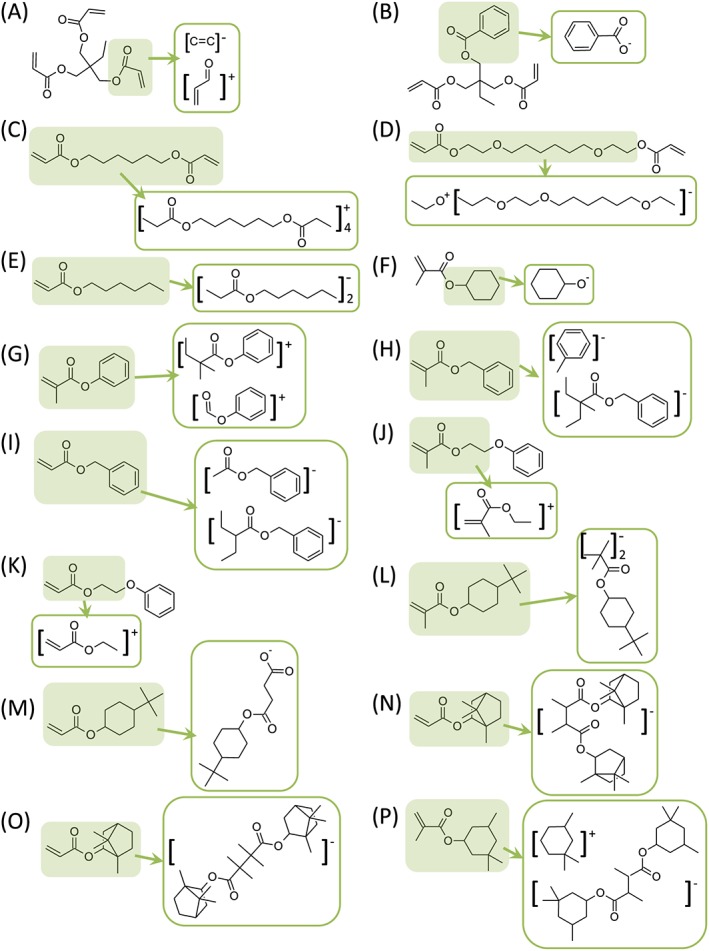
Chemical structures of the 16 monomers (A–P) along with the most unique ions for each material (ions with the highest *Ψ* value).

In addition to the SIMS ions that were most intense for a particular polymer, ions that were unique for a given material were also investigated. To identify the unique ions for each polymer, the intensity of a given ion was divided by the largest intensity of that ion observed for any of the other 15 materials, denoted as *Ψ*. This was conducted for each ion of every polymer. The highest values of *Ψ* for each polymer were identified and are listed in Table SI2. Ions were considered as unique where *Ψ* > 2, such that the intensity of a particular ion for a material was twice that of any other material. For polyM, no ions were identified where *Ψ* > 2, and in these cases, the ions where *Ψ* > 1 are presented. A summary of the ions with the highest values of *Ψ* are shown in Fig. 5.

A number of the unique ions identified for materials, including polymers of monomers C, E, F, I, L, N, O and P, were relatively large (*m*/*z* > 200) and likely originated from dimers or trimers of the monomer unit (Fig. 4). For example, the C_18_H_30_O_4_^+^ ion derived from a dimer of monomer G was characteristic for polyG. The prevalence of large ions within those ions specific to materials was unsurprising considering the similarities in the materials used in the study, thus, requiring larger fragments to differentiate the materials.

A number of polymers produced unique ions that were derived from both the acrylate/methacrylate group and the pendant group. For acrylate/methacrylate homologues, the distinguishing chemistry, and therefore a characteristic ion would likely include the polymerisable group and the pendant group. For example, the C_6_H_9_O_2_^+^ and C_5_H_7_O_2_^+^ ions were found to be characteristic of the methacrylate/acrylate pair of polyJ and polyK, respectively, whereby the ions corresponded to each of the monomer units with the loss of a phenyl group (Fig. 4(J and K)).

The unique ions for polyA were associated with the acrylate group, such as C_2_H_3_^−^ and C_3_H_4_O^+^ (Fig. 4(A)). Because of the relatively large number of acrylate groups on this monomer and the increased likelihood that polyA would contain unreacted acrylate moieties, it was unsurprising that this functional group differentiates polyA from the other 15 polymers.

### Principal component analysis

In order to assess the major variance within the SIMS data for the 16 materials used in this study, PCA was applied. Based upon the Eigenvalue plot (Fig. SI3), nine PC were selected to describe the variance within the dataset. A summary of each PC is presented in Fig. SI4–12.

Principal component 1 (Fig. SI4) accounted for 20% of the variance within the entire dataset. Ions with positive loadings for PC1 were characteristic of benzene, such as C_7_H_7_^+^ and C_6_H_5_O^−^ (Fig. 5(B)). The materials with positive scores for PC1 were all mono(meth)acrylates with benzene pendant groups (Fig. SI4(A)), therefore, the largest observed variance identified in PCA for the materials used in this study was the discrimination of the benzene group. PolyF was also assigned a positive score for PC1 despite containing no benzene groups. This is likely due to the removal of hydrogen groups from the cyclohexyl group during the ionisation process. The positive score for PC1 is, thus, associated with the presence of a benzene group or a benzene precursor. For PolyF, the presence of unsaturated C_6_ fragments within its ToF‐SIMS spectrum indicated that the polymer's pendant groups are cyclohexane rather than benzene. It is noted that in addition to ions associated with benzene, there were also ions associated with the methacrylate group (C_4_H_5_O_2_^−^ and C_4_H_5_O^+^) that were assigned positive loadings for PC1, whilst ions associated with the acrylate group (C_3_H_3_O_2_^−^) were assigned negative loadings (Fig. SI4(C)). The convolution of benzene pendant groups and backbone chemistry within the variance captured by PC1 is likely due to the presence of more methacrylate monomers with benzene groups than acrylate monomers within the library studied. This observation highlights the importance of understanding the chemical variance of the materials in the study and the behaviour of various functional groups resulting from ToF‐SIMS analysis in order to correctly interpret PCA results. The prominence of benzyl groups within PC1 was likely due to the high frequency of benzyl fragments within the SIMS spectra because of resonance stabilisation.^42^ It is important to note that the most stable ion fragments will be predominant within the SIMS data when assessing the importance of the variance captured by a particular PC.

**Figure 6:**
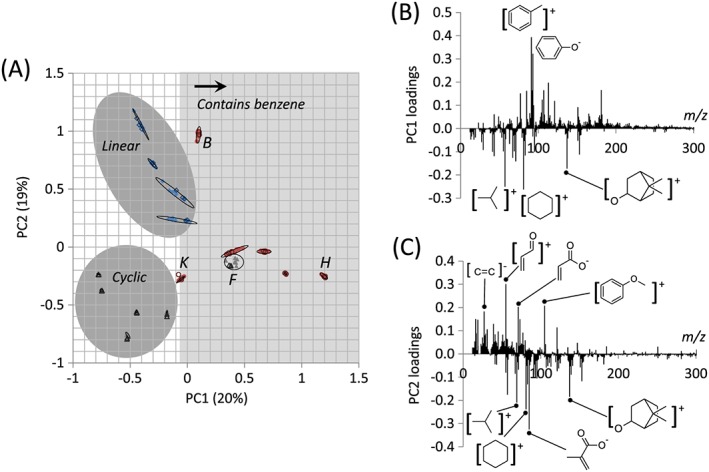
(A) Scores plot for PC 1 and 2 for the 16 polymers. Labels denote the monomer used to prepare the polymer, including monomers with linear (◆), cyclic (▲) or aromatic (●) pendant groups for both the training (filled) and test (open) datasets. (B and C) Loadings plot for PC 1 (B) and 2 (C). Ellipses depicting 95% confidence limits for each polymer have been drawn.

The separation of the mono‐functional and multi‐functional (meth)acrylates was observed for PC2 (Fig. SI5), which represented 19% of the variance within the dataset. The ions with positive loadings for PC2 included those characteristic of polyB, such as C_7_H_5_O^+^ and C_7_H_5_O_2_^−^, as well as the C_3_H_3_O^+^ ion that likely originated from unreacted acrylate (Fig. 6(C), Fig. SI5(C)). Ions assigned with negative loadings for PC2 were found to be a characteristic of polymers of monomers l‐P, such as the C_4_H_9_^+^ ion originating from the *tert*‐butyl group on polyL and polyM, the C_9_H_13_O^+^ ion derived from the isobornyl pendant group of polyN and polyO and the C_6_H_9_^+^ ion that was characteristic of polyM and N's pendant groups (Fig. SI5(C)). Additionally, the methacrylate ion, C_4_H_5_O_2_^−^, was assigned negative loadings for PC2, and in this case was associated with mono‐functional methacrylates as no multi‐functional methacrylates were used in this study.

Together, PC1 and PC2 separated linear, cyclic and aromatic pendant groups within the polymer library (Fig. 6(A)). Replicate samples clustered together demonstrating reproducibility of the sample chemistry. Both training and test sets also clustered, demonstrating that the PCA robustly captured the variance within the dataset and that the identity of the test set polymer samples could be successfully identified by reference to the scores plots of known samples (Fig. 6(A)).

**Figure 7:**
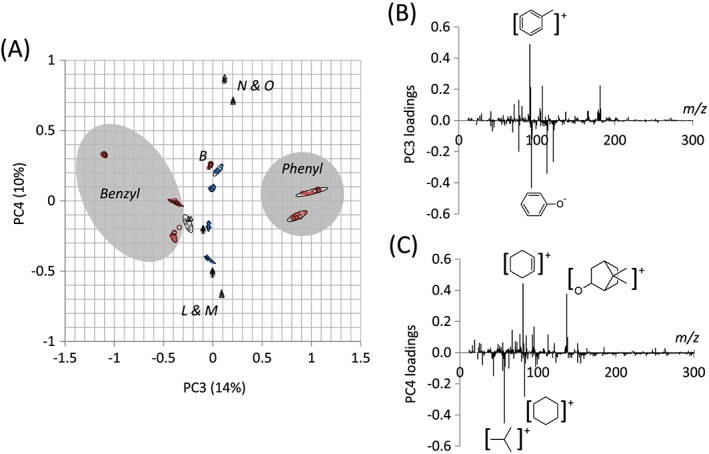
(A) Scores plot for PC 3 and 4 for the 16 polymers, including monomers with linear (◆), cyclic (▲) or aromatic (●) pendant groups for both the training (filled) and test (open) datasets. (B and C) Loadings plot for PC 3 (B) and 4 (C). Ellipses depicting 95% confidence limits for each polymer have been drawn.

A total of 14% of the variance within the dataset was represented by PC3 (Fig. SI6), which was also associated with benzene groups, differentiating benzyl and phenyl functional materials. This is demonstrated by the large positive loading of the C_7_H_7_^+^ ion characteristic of the benzyl group and the large negative loading of the C_6_H_5_O^−^ ion, characteristic of the phenyl group (Fig. 7(B)).

The 10% variance captured by PC4 was associated with differences in the saturated pendant groups within the polymer library, in particular, separating the isobornyl acrylate/methacrylate pair of polyN and polyO from the *tert*‐butyl cyclohexyl acrylate/methacrylate pair of polyL and polyM (Fig. SI7). The ions C_6_H_9_^+^ and C_9_H_13_O^+^ associated with the isobornyl group were assigned positive loadings for PC4 whilst the C_4_H_9_^+^ and C_6_H_11_^+^ ions associated with the *tert*‐butyl cyclohexyl group were assigned negative loadings for PC4 (Fig. 7(C)).

Together, PC3 and PC4 differentiate cyclic and aromatic pendant groups, with the aromatic group containing monomers being separated as phenyl or benzyl, and the cyclic group containing monomer being separated as isobornyl or *tert*‐butyl cyclohexyl (Fig. 7(A)).

Principal component 6 represented 7% of the variance within the dataset (Fig. SI9). All the materials with positive scores for PC6 were polymethacrylates, whilst those with negative scores were polyacrylates. The C_4_H_5_O_2_^−^ ion, characteristic of methacrylate groups, was assigned with positive loadings whilst the C_3_H_3_O_2_^−^ ion, characteristic of acrylate groups, was assigned with negative loadings (Fig. SI9(C)). Other ions that also featured prominently in PC6 were unique to specific materials, such as the C_8_H_13_O_2_^−^ ion characteristic of polyP, the C_5_H_5_O^−^ ion characteristic of polyI and the C_6_H_9_^+^ and C_9_H_13_O^+^ ions characteristic of polyN and polyO. These pendant groups were, thus, identified as being associated with either the methacrylate or acrylate monomers featured within the monomer library used in this study. It is noted that some ions have higher negative loadings than the ions characteristic of the acrylate group and, thus, are more influential in the separation of the samples. As such, the variance captured by PC6 is representative of a number of chemical differences.

The 6% variance captured by PC7 identified differences with the di/triacrylates (Fig. SI10). Ions characteristic of the benzoic acid group on polyB, such as C_7_H_5_O^+^ and C_7_H_5_O_2_^−^, were assigned positive loadings whilst short fragments likely derived from the fragmentation of the ester group, such as C_2_H_5_O^+^ and CH_3_O^+^, were assigned negative loadings (Fig. SI10(C)). These short fragments were characteristic of the di/triacrylates as these monomers had a higher number of ester groups than monoacrylates. Additionally, polyA, polyC and polyD could not readily form ions by the breaking of a single bond, and consequently, their SIMS spectra were richer in ions derived from breaking two or more bonds.^61^

The remaining three PCs (5, 8 and 9) together captured 16% of the variance within the dataset and were associated with the different pendant groups within the monomer library. Ions associated with benzene such as the C_6_H_9_O_2_^+^, C_6_H_5_^+^ and C_7_H_7_^+^ ions were assigned positive loadings for PC5, whilst the C_10_H_15_O^+^, C_6_H_11_O^−^ and C_8_H_11_O_2_^−^ ions associated with large saturated ring fragments were assigned negative loadings for PC5. PC8 was also associated with differences in ring structures, with ions C_9_H_9_O_2_^+^ and C_8_H_13_O_2_^−^ being assigned positive loadings and ions C_6_H_5_O^−^ and C_9_H_13_O_2_^−^ being assigned negative loadings. PC9 captures variance associated with both pendant groups and backbone structure with the methacrylate/acrylate ions C_4_H_5_O_2_^−^ and C_3_H_3_O_2_^−^ both having large positive or negative loadings, respectively, whilst the C_3_H_7_^+^ ion originating from linear hydrocarbon groups and ions C_6_H_9_O_2_^−^ and C_6_H_6_O^−^ associated with benzene containing groups also had large positive or negative loadings for this PC.

In this study, the prior assessment of the intense and unique ions for each material assisted in the interpretation of the principal component and facilitated the identification of the chemical variance represented by each component. In particular, the PCs had large loadings for the most intense ions within the spectra being assessed and as such assigning the likely origins of these ions prior to PCA assisted with interpreting the variance captured by the various PCs. The laterally resolved, large area scanning capabilities of ToF‐SIMS applied to the microarray format allowed SIMS spectra from 160 unique materials to be rapidly acquired. This large amount of data permitted chemically diverse materials to be compared with a high number of replicates, allowing for trends to be identified and robustly assessed.

### Principal component analysis applied to copolymers

**Figure 8:**
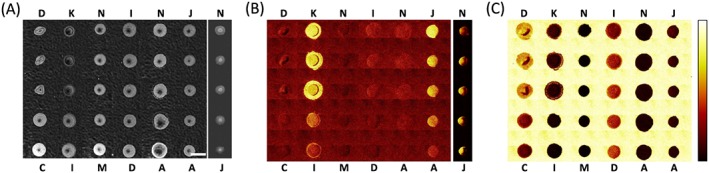
Assessment of the copolymer microarray produced. Monomers used to for copolymer series are indicated for each column and from top to bottom are for monomer ratios of 4 : 1, 3 : 2, 1 : 1, 2 : 3 and 1 : 4. (A) Mosaic of phase contrast images of the polymer microarray, scale bar = 500 µm. To the right of the main image, the phase contrast image of the copolymer series of monomers *j* and *n* printed onto glass is shown. (B and C) ToF‐SIMS ion images from the microarray area corresponding to (B) C_6_H_5_^+^ and (C) C_2_H_5_O^+^. Images shown at same scale as (A). Intensity scale for (B) and (C) shown to the right of (C). (B) To the right of the main image, the ion image of the copolymer series of monomers *j* and *n* printed onto glass is shown.

Monomer pairs of *c* and *d*, *i* and *k*, *m* and *n*, *d* and *i*, *a* and *n*, *a* and *j*, and *j* and *n* were prepared as a second microarray at ratios of 4 : 1, 3 : 2, 1 : 1, 2 : 3 and 1 : 4 in triplicate and assessed by phase contrast microscopy and ToF‐SIMS. Images of the array are shown in Fig. 8. All copolymers were successfully printed and remained distinct from each other. Because of spreading on pHEMA, the copolymer pair of monomers *j* and *n* was instead printed onto bare glass. The intensity of the ion C_6_H_5_^+^ was observed to increase for the copolymer pairs of monomers *k* and *i*, and *j* and *a* as monomers *k* or *j* were increased (Fig. 8(B)). A high intensity of the ion C_6_H_5_^+^ was previously observed for polyK and polyJ compared with polyA and polyI (Fig. 3), therefore, it is expected that this ion would correlate with the monomer content of monomers *k* and *j* for these copolymer pairs.

**Figure 9:**
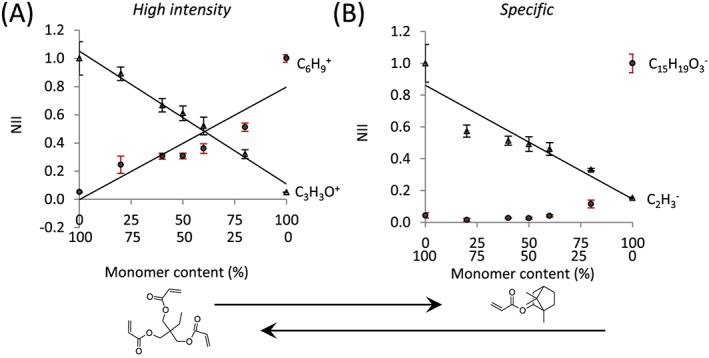
Normalised ion intensity for (A) high intensity ions and (B) specific ions for polyA and polyN across the copolymer of monomers *a* and *n*. Ions representative of polyA were (A) C_3_H_3_O^+^ and (B) C_2_H_3_^−^ (△), whilst for polyN were (A) C_6_H_9_^+^ and (B) C_15_H_19_O_3_^−^ (●). The peak list determined from the homopolymer library was applied to each polymer and used to determine the total counts used to normalise the ion intensities. Ion intensity for representative ions was then further normalised to the intensity of the respective homopolymer. Error bars equal ± one standard deviation unit, *n* = 10 for homopolymers and *n* = 3 for copolymers. Lines of best fit are shown, *R* ^2^ = 0.98 for C_3_H_3_O^+^, 0.83 for C_6_H_9_^+^ and 0.88 for C_2_H_3_^−^.

To assess changes in surface chemistry across the seven copolymer series, a high intensity ion and a specific ion for each homopolymer were selected from previous analysis (Figs 3 and 5), and the normalised ion intensity was plotted for the various monomer compositions (Fig. SI13). In order to quantitatively compare the homopolymers and copolymers, the total counts from the ions found within the peak list determined from the homopolymer library was used to normalise the ion intensities. For all copolymer pairs except for monomers *c* and *d*, the normalised ion intensity of representative ions for a copolymer series varied linearly with monomer composition. For the copolymer series of monomers *c* and *d*, the chemical similarity of the two monomers and cross‐linked nature of the polymers prevented a clear correlation between ion intensity and bulk composition being identified. As an example, the normalised ion intensity for high intensity ions and specific ions for the copolymer pair of monomers *a* and *n*, shown in Fig. 9, varied linearly with copolymer composition for ions C_3_H_3_O^+^ (*R* ^2^ = 0.98, high intensity ion for polyA) and C_6_H_9_^+^ (*R* ^2^ = 0.83, high intensity ion for polyN) suggesting that the surface chemistry of the copolymer series was representative of the bulk composition and no surface segregation of a particular monomer was evident.^6^ The ion C_2_H_3_^−^, specific to polyA, varied linearly with monomer composition (*R* ^2^ = 0.88); however, the ion C_15_H_19_O_3_^−^, specific for polyN, did not vary linearly with monomer composition (*R* ^2^ = 0.47), with its intensity decreasing to a baseline level as the content of monomer *a* was increased from 0% to 40%. The ion C_15_H_19_O_3_^−^ likely originated from a dimer of monomer *n* and, therefore, a high intensity of this ion would be expected for polyN where a high frequency of two adjacent monomer *n* units would occur. However, the rapid decrease in ion intensity with increasing monomer *n* content suggested that at the surface of the polymer, monomer *n* preferentially bound to a monomer *a* unit rather than to another monomer *n* unit, and as such, the monomer sequence would likely be alternating rather than a random or block sequence. This demonstrates that the SIMS analysis was able to provide insight into the molecular structure of a copolymer series. The cross‐linked nature of this copolymer series means molecular reorientations subsequent to polymerisation is unlikely and therefore, as surface segregation of monomers prior to polymerisation has already been excluded, the surface chemistry is likely representative of the bulk composition.

Scores for PC 1 to 4 were calculated for the ToF‐SIMS data from the copolymer library as a test set and compared with the homopolymer counterparts. To allow this comparison, the same peak lists were applied to both datasets, although the peak list was initially determined from the homopolymer library, and thus did not contain any peaks unique to the copolymer set. To allow comparison, the datasets were normalised to the total counts from those peaks included within the peak list rather than the total ion count. Scores plots for PC1–PC4 for each copolymer series are shown in Fig. SI14.

**Figure 10:**
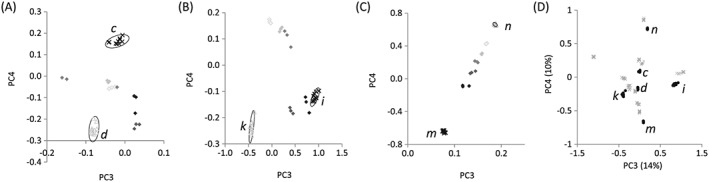
Scores plot of PC3 and PC4 for copolymers of monomers (A) *m* and *n*, (B) *i* and *k* and (C) *c* and *d* at ratios of 1 : 0 (×), 4 : 1 (◆), 3 : 2 (◆), 1 : 1 (◆), 2 : 3 (◆), 1 : 4 (◇) and 0 : 1 (△). Ten repeats for homopolymers and three repeats for each copolymer are plotted. Ellipses depicting 95% confidence limits for the homopolymers. (D) Scores plots for PC3 and PC4 for all 16 homopolymers. PolyC, polyD, polyI, polyK, polyM and polyN are highlighted as ‘●’.

In 15 of the 28 cases, a correlation (*R* ^2^ > 0.75) was observed between monomer composition and the PC score for the seven copolymer series and PCs 1 to 4, with at least one correlation observed for each copolymer series (Table 1). For example, the scores plot of the copolymers of monomers *m* and *n* for PC3 and PC4 (Fig. 10(A)) linearly correlated with monomer composition (*R* ^2^ = 0.96 for both PCs). As such, PC3 and PC4 captured the chemical variance across this copolymer series and could be used to predict the chemical composition of an unknown copolymer derived from monomers *m* and *n*. For the copolymer series of monomers *k* and *i* (Fig. 10(B)), the scores for PC3 correlated with monomer compositions (*R* ^2^ = 0.99), whereas PC4 did not (*R* ^2^ = 0.01). No correlation was observed for either PC for the copolymer series of monomers *c* and *d* (PC3, *R* ^2^ = 0.37, PC4, *R* ^2^ = 0.69, Fig. 10(C)). The failure of a particular PC to correlate with the chemical composition across a copolymer series was often observed when the differences in the scores of the respective homopolymers was small compared with the total score variance observed for all copolymers (Fig. 10(D)).

**Table 1:**
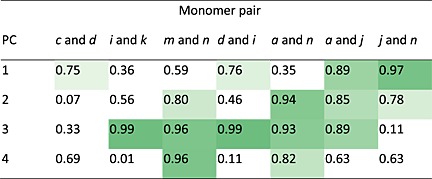
Correlation (*R* ^2^ value) of monomer composition with principal component score for seven copolymer pairs.

*R* ^2^ values >0.75 have been shaded according to their value.

## Conclusions

In this study, a polymer microarray containing a series of 16 polymers that contained C_6_ derived pendant groups was used as an example of a multicomponent system. The microarray was analysed by ToF‐SIMS, and the hyperspectral datasets were analysed. Initially, the most intense and unique ions for each polymer were identified and, using the polymer structures, assessed for their likely chemical origin. Ring structures were observed to be more stable and less fragmented by the SIMS ionisation process than their linear counterparts. Consistent with previous studies, the resonance stabilised benzene ion was predominant in SIMS spectra for materials with benzyl pendant groups and was also observed for materials containing cyclohexyl pendant groups. The SIMS spectra from multi‐functional monomers were characterised by ions derived from the acrylate group as well as short hydrocarbon fragments, likely due to the number of acrylates and the overall stability of the resultant cross‐linked polymer.

Principal component analysis was used to assess the variance between the SIMS spectra for the 16 poly(meth)acrylates. The largest variance was associated with the presence of a benzene group, likely due to the stability and thus high frequency of benzyl ions. Other variance captured by the PCA in order of the amount of variance represented included distinguishing mono‐functional from multi‐functional monomers, phenyl from benzyl groups, various saturated cyclic pendant groups and acrylates from methacrylates. PCs 1 to 4 were applied to seven copolymer series as test sets, whereupon a correlation (*R* ^2^ > 0.75) was observed between PC score and monomer composition in 15 of the 28 cases. The chemical variance captured by the PCs for the homopolymer set was, thus, predictive for the composition of associated copolymers. In some cases, assessing the SIMS spectra across a copolymer series provided insight into the molecular sequence of the copolymer. The robust interpretation of the SIMS data presented in this study and the insights therein gained are useful to interpret multivariate analysis of SIMS for polymeric systems and offer a guide for assessing complex hyperspectral datasets generally. Polymer microarrays significantly enhanced the scope of this study and will likely continue to be an important sample format for studying ToF‐SIMS analysis of material systems.

## Supporting information

Supporting Information

Supporting Information
