# Supplementary material for: ToF‐SIMS analysis of a polymer microarray composed of poly(meth)acrylates with C6 derivative pendant groups
Source: Surf Interface Anal. 2016 Feb 19;48(4):226–36. doi: 10.1002/sia.5959 (PMC4832844; doi:10.1002/sia.5959)
Supplement: Supplementary file 2 — Supporting Information [file SIA-48-226-s002.docx]

**ToF-SIMS analysis of a polymer microarray composed of poly(meth)acrylates with C_6_ derivative pendant groups – Supporting information**

Andrew L. Hook*, David J. Scurr

*corresponding author

andrew.hook@nottingham.ac.uk

Laboratory of Biophysics and Surface Analysis

University of Nottingham

Nottingham, NG7 2RD, UK

**Description of PeakFinder algorithm**

To identify chemical assignments for a given mass the PeakFinder algorithm sequentially built possible chemical structures, calculated the deviation from the target mass, and recorded the possible assignment if it fell within the user defined deviation limit. Chemical structures were initially built by calculating the total possible carbon atoms then sequentially adding hydrogen, fluorine, miscellaneous elements, oxygen, nitrogen and silicon. The number of carbons was then reduced by one and the process was continued until chemical structures with no carbon were considered. At all times the amount of each element was restricted to user defined limits. The amount of unpaired electrons within each structure was calculated. Where possible, unpaired electrons were paired to introduce unsaturation or cyclic structures. The total allowable amount of unsaturation was user defined. A negative ion was considered to reduce the amount of unpaired electrons by one whereas a positive ion was considered to increase the amount of unpaired electrons by one. Potential assignments that were outside the user defined allowable number of unpaired electrons were excluded. All possible assignments for a mass were listed (ranked from lowest deviation to highest deviation) along with the deviation (ppm) from the mass and a valence error (determined from the number of unpaired electrons and the degree of unsaturation).

**Table SI1.** The highest intensity ions for each material with the corresponding ion intensity. Error range given equals ± 1 standard deviation, n = 10.

| A | | | B | | | C | | | D | | | E | | | F | | | G | | | H | | |
| --- | --- | --- | --- | --- | --- | --- | --- | --- | --- | --- | --- | --- | --- | --- | --- | --- | --- | --- | --- | --- | --- | --- | --- |
| Assignment | Ion intensity | | Assignment | Ion intensity | | Assignment | Ion intensity | | Assignment | Ion intensity | | Assignment | Ion intensity | | Assignment | Ion intensity | | Assignment | Ion intensity | | Assignment | Ion intensity | |
| C_3_H_3_O_2_^-^ | 0.120 | ± 0.012 | C_7_H_5_O^+^ | 0.177 | ± 0.008 | C_3_H_5_^+^ | 0.110 | ± 0.004 | C_3_H_7_^+^ | 0.090 | ± 0.005 | C_3_H_7_^+^ | 0.183 | ± 0.013 | C_6_H_5_O^-^ | 0.150 | ± 0.014 | C_6_H_5_O^-^ | 0.193 | ± 0.007 | C_7_H_7_^+^ | 0.254 | ± 0.006 |
| C_3_H_5_^+^ | 0.097 | ± 0.006 | C_6_H_5_^+^ | 0.116 | ± 0.007 | C_2_H^-^ | 0.083 | ± 0.001 | C_3_H_5_^+^ | 0.081 | ± 0.003 | C_7_H_5_O^+^ | 0.077 | ± 0.007 | C_4_H_5_O_2_^-^ | 0.080 | ± 0.014 | C_7_H_7_^+^ | 0.072 | ± 0.007 | C_4_H_5_O_2_^-^ | 0.191 | ± 0.01 |
| C_3_H_3_O^+^ | 0.096 | ± 0.012 | C_2_H^-^ | 0.095 | ± 0.002 | C_4_H_7_^+^ | 0.059 | ± 0.001 | C_2_H^-^ | 0.063 | ± 0.003 | C_2_HO^-^ | 0.057 | ± 0.002 | C_3_H_5_^+^ | 0.077 | ± 0.006 | C_7_H_5_O^+^ | 0.070 | ± 0.008 | C_2_H^-^ | 0.060 | ± 0.001 |
| C_2_H^-^ | 0.082 | ± 0.005 | C_7_H_5_O_2_^-^ | 0.065 | ± 0.002 | O^-^ | 0.050 | ± 0.001 | C_2_HO^-^ | 0.058 | ± 0.002 | C_3_H_5_^+^ | 0.057 | ± 0.002 | C_4_H_7_^+^ | 0.074 | ± 0.004 | C_5_H_9_^+^ | 0.059 | ± 0.003 | C_7_H_7_O^-^ | 0.052 | ± 0.003 |
| C_2_H_3_^+^ | 0.061 | ± 0.005 | C_3_H_5_^+^ | 0.062 | ± 0.001 | C_2_H_3_^+^ | 0.049 | ± 0.002 | C_4_H_7_^+^ | 0.053 | ± 0.002 | C_4_H_7_^+^ | 0.050 | ± 0.003 | C_7_H_5_O^+^ | 0.068 | ± 0.008 | C_2_H^-^ | 0.055 | ± 0.002 | C_7_H_8_^+^ | 0.048 | ± 0.001 |
| C_3_H_3_^+^ | 0.050 | ± 0.004 | C_3_H_3_O_2_^-^ | 0.061 | ± 0.002 | C_3_H_3_^+^ | 0.047 | ± 0.002 | C_6_H_5_O^-^ | 0.044 | ± 0.011 | C_6_H_5_O^-^ | 0.047 | ± 0.013 | C_7_H_7_^+^ | 0.048 | ± 0.003 | C_4_H_5_O_2_^-^ | 0.052 | ± 0.005 | C_6_H_5_O^-^ | 0.041 | ± 0.016 |
| O^-^ | 0.048 | ± 0.003 | C_6_H_5_^-^ | 0.047 | ± 0.001 | C_2_HO^-^ | 0.046 | ± 0.001 | C_2_H_5_O^+^ | 0.043 | ± 0.004 | C_3_H_3_O_2_^-^ | 0.043 | ± 0.003 | C_2_H^-^ | 0.045 | ± 0.001 | C_3_H_5_^+^ | 0.050 | ± 0.002 | C_11_H_17_O_2_^+^ | 0.035 | ± 0.001 |
| C_2_H_3_^-^ | 0.045 | ± 0.005 | C_3_H_3_O^+^ | 0.042 | ± 0.003 | C_2_H_5_^+^ | 0.042 | ± 0.002 |  |  |  | C_2_H^-^ | 0.036 | ± 0.001 | C_9_H_11_O_2_^+^ | 0.036 | ± 0.006 | C_6_H_5_^+^ | 0.045 | ± 0.003 |  |  |  |
| C_2_HO^-^ | 0.045 | ± 0.002 | C_4_H^-^ | 0.041 | ± 0.002 | C_3_H_7_^+^ | 0.041 | ± 0.002 |  |  |  | C_9_H_11_O_2_^+^ | 0.029 | ± 0.003 | C_5_H_9_^+^ | 0.030 | ± 0.003 |  |  |  |  |  |  |
| C_2_H_5_^+^ | 0.037 | ± 0.003 |  |  |  | OH^-^ | 0.038 | ± 0.001 |  |  |  | C_2_H_5_^+^ | 0.026 | ± 0.002 | C_6_H_9_^+^ | 0.029 | ± 0.002 |  |  |  |  |  |  |
|  |  |  |  |  |  | C_3_H_3_O^+^ | 0.035 | ± 0.002 |  |  |  | C_7_H_7_^+^ | 0.025 | ± 0.005 | C_6_H_11_^+^ | 0.028 | ± 0.001 |  |  |  |  |  |  |
|  |  |  |  |  |  | C_6_H_5_^+^ | 0.032 | ± 0.001 |  |  |  |  |  |  |  |  |  |  |  |  |  |  |  |
| I | | | J | | | K | | | L | | | M | | | N | | | O | | | P | | |
| Assignment | Ion intensity | | Assignment | Ion intensity | | Assignment | Ion intensity | | Assignment | Ion intensity | | Assignment | Ion intensity | | Assignment | Ion intensity | | Assignment | Ion intensity | | Assignment | Ion intensity | |
| C_7_H_7_^+^ | 0.235 | ± 0.012 | C_6_H_5_O^-^ | 0.219 | ± 0.003 | C_6_H_5_O^-^ | 0.156 | ± 0.012 | C_4_H_9_^+^ | 0.188 | ± 0.003 | C_4_H_9_^+^ | 0.226 | ± 0.004 | C_6_H_9_^+^ | 0.179 | ± 0.005 | C_4_H_5_O_2_^-^ | 0.170 | ± 0.008 | C_5_H_9_^+^ | 0.190 | ± 0.004 |
| C_2_HO^-^ | 0.080 | ± 0.003 | C_4_H_5_O_2_^-^ | 0.126 | ± 0.005 | C_4_H_9_^+^ | 0.133 | ± 0.009 | C_4_H_5_O_2_^-^ | 0.138 | ± 0.008 | C_2_HO^-^ | 0.079 | ± 0.002 | C_2_HO^-^ | 0.087 | ± 0.002 | C_6_H_9_^+^ | 0.169 | ± 0.005 | C_4_H_5_O_2_^-^ | 0.141 | ± 0.01 |
| C_4_H_9_^+^ | 0.073 | ± 0.006 | C_6_H_5_^+^ | 0.126 | ± 0.003 | C_6_H_5_^+^ | 0.061 | ± 0.002 | C_5_H_9_^+^ | 0.074 | ± 0.000 | C_3_H_5_^+^ | 0.078 | ± 0.001 | C_3_H_3_O_2_^-^ | 0.077 | ± 0.002 | C_9_H_13_O^+^ | 0.085 | ± 0.002 | C_4_H_7_^+^ | 0.080 | ± 0.001 |
| C_3_H_3_O_2_^-^ | 0.066 | ± 0.001 | C_6_H_9_O_2_^+^ | 0.096 | ± 0.002 | C_2_HO^-^ | 0.054 | ± 0.001 | C_3_H_5_^+^ | 0.069 | ± 0.001 | C_5_H_9_^+^ | 0.073 | ± 0.001 | C_5_H_9_^+^ | 0.073 | ± 0.000 | C_5_H_9_^+^ | 0.074 | ± 0.001 | C_3_H_5_^+^ | 0.071 | ± 0.001 |
| C_2_H^-^ | 0.062 | ± 0.001 | C_8_H_9_O^+^ | 0.074 | ± 0.002 | C_2_H^-^ | 0.053 | ± 0.001 | C_6_H_11_^+^ | 0.069 | ± 0.001 | C_6_H_11_^+^ | 0.069 | ± 0.001 | C_2_H^-^ | 0.069 | ± 0.001 | C_2_H^-^ | 0.061 | ± 0.001 | C_6_H_11_^+^ | 0.053 | ± 0.001 |
| C_5_H_5_O^-^ | 0.033 | ± 0.001 | C_2_H^-^ | 0.062 | ± 0.001 | C_5_H_9_^+^ | 0.051 | ± 0.001 | C_4_H_7_^+^ | 0.057 | ± 0.000 | C_3_H_3_O_2_^-^ | 0.065 | ± 0.001 | C_5_H_7_^+^ | 0.061 | ± 0.001 | C_5_H_7_^+^ | 0.056 | ± 0.001 | C_2_H^-^ | 0.048 | ± 0 |
| C_3_H_5_^+^ | 0.033 | ± 0.002 | C_5_H_9_^+^ | 0.060 | ± 0.001 | C_3_H_5_^+^ | 0.048 | ± 0.001 | C_2_H^-^ | 0.056 | ± 0.001 | C_4_H_7_^+^ | 0.063 | ± 0.000 | C_9_H_13_O^+^ | 0.060 | ± 0.002 | C_3_H_5_^+^ | 0.051 | ± 0.001 |  |  |  |
| C_5_H_9_^+^ | 0.032 | ± 0.002 | C_3_H_5_^+^ | 0.050 | ± 0.001 | C_4_H_5_O_2_^-^ | 0.045 | ± 0.003 | C_3_H_7_^+^ | 0.044 | ± 0.000 | C_2_H^-^ | 0.061 | ± 0.001 | C_3_H_5_^+^ | 0.056 | ± 0.001 | C_3_H_7_^+^ | 0.042 | ± 0.001 |  |  |  |
|  |  |  |  |  |  | C_6_H_11_^+^ | 0.044 | ± 0.002 |  |  |  | C_3_H_7_^+^ | 0.051 | ± 0.001 | C_3_H_7_^+^ | 0.047 | ± 0.000 | C_7_H_11_^+^ | 0.040 | ± 0.000 |  |  |  |
|  |  |  |  |  |  |  |  |  |  |  |  |  |  |  | C_7_H_11_^+^ | 0.044 | ± 0.001 |  |  |  |  |  |  |

**Table SI2.** Unique ions for each material, where the factor Ψ = the intensity of the ion for the material listed divided by highest intensity observed for that ion for any of the other materials.

| A | | B | | C | | D | | E | | F | | G | | H | |
| --- | --- | --- | --- | --- | --- | --- | --- | --- | --- | --- | --- | --- | --- | --- | --- |
| Assignment | Ψ | Assignment | Ψ | Assignment | Ψ | Assignment | Ψ | Assignment | Ψ | Assignment | Ψ | Assignment | Ψ | Assignment | Ψ |
| C_2_H_3_^-^ | 2.9 | C_7_H_5_O_2_^-^ | 5.2 | C_40_H_62_O_16_^-^ | 2.0 | C_2_H_2_O^-^ | 8.1 | C_17_H_29_O_4_^-^ | 4.7 | C_6_H_9_O^-^ | 4.9 | C_12_H_12_O_2_^+^ | 3.0 | C_14_H_17_O_2_^-^ | 6.1 |
| C_2_H_4_^-^ | 2.8 | C_7_H_6_O_2_^-^ | 3.6 |  |  | C_13_H_15_O_3_^-^ | 4.6 | C_19_H_20_O_4_^-^ | 3.6 | C_31_H_41_O_7_^-^ | 3.8 | C_7_H_8_O_2_^+^ | 2.8 | C_7_H_7_^-^ | 2.7 |
| C_3_H_4_O^+^ | 2.3 | C_6_H_4_^-^ | 2.7 |  |  | C_2_H_5_O^+^ | 4.0 | C_11_H_11_O^-^ | 3.5 | C_31_H_42_O_7_^-^ | 2.8 | C_42_H_55_O_8_^-^ | 2.4 | C_12_H_21_O^+^ | 2.4 |
| C_3_H_3_O^+^ | 2.3 | C_6_H_4_^+^ | 2.3 |  |  | C_3_H_6_^-^ | 2.8 | C_45_H_76_O_8_^-^ | 3.3 | C_47_H_53_O_9_^-^ | 2.7 | C_18_H_30_O_4_^+^ | 2.4 | C_12_H_22_O^+^ | 2.1 |
|  |  | C_7_H_5_O^+^ | 2.3 |  |  | C_2_H_6_O^+^ | 2.6 | C_13_H_7_O_2_^-^ | 3.1 | C_19_H_29_O_4_^+^ | 2.5 | C_7_H_8_O^-^ | 2.3 |  |  |
|  |  |  |  |  |  | C_13_H_16_O_3_^-^ | 2.3 | C_10_H_6_O_2_^-^ | 2.8 | C_41_H_37_O_4_^-^ | 2.3 | C_11_H_13_O^+^ | 2.1 |  |  |
|  |  |  |  |  |  | C_2_H_2_O_2_^-^ | 2.1 | C_29_H_27_O_6_^-^ | 2.8 | C_6_H_10_O^-^ | 2.3 | C_24_H_45_O_6_^+^ | 2.1 |  |  |
|  |  |  |  |  |  |  |  | C_28_H_40_O_6_^-^ | 2.7 | C_18_H_25_O_4_^+^ | 2.1 | C_12_H_8_O_2_^-^ | 2.1 |  |  |
|  |  |  |  |  |  |  |  |  |  |  |  | C_14_H_14_O_3_^-^ | 2.0 |  |  |
|  |  |  |  |  |  |  |  |  |  |  |  | C_15_H_18_O_3_^-^ | 2.0 |  |  |
| I | | J | | K | | L | | M | | N | | O | | P | |
| Assignment | Ψ | Assignment | Ψ | Assignment | Ψ | Assignment | Ψ | Assignment | Ψ | Assignment | Ψ | Assignment | Ψ | Assignment | Ψ |
| C_20_H_21_O_3_^-^ | 2.6 | C_6_H_9_O_2_^+^ | 16.5 | C_5_H_7_O_2_^+^ | 4.4 | C_27_H_25_O_4_^-^ | 11.1 | C_14_H_17_O_4_^-^ | 1.5 | C_26_H_25_O_4_^-^ | 2.8 | C_28_H_29_O_4_^-^ | 6.1 | C_26_H_23_O_4_^-^ | 17.8 |
| C_9_H_9_O_2_^-^ | 2.2 | C_6_H_10_O_2_^+^ | 6.1 |  |  | C_40_H_45_O_7_^-^ | 6.3 | C_14_H_18_O_4_^-^ | 1.2 | C_27_H_30_O_3_^-^ | 2.8 | C_27_H_42_O_4_^-^ | 5.1 | C_8_H_13_O_2_^-^ | 17.4 |
| C_13_H_13_O_2_^-^ | 2.2 | C_8_H_9_O^+^ | 2.4 |  |  | C_25_H_34_O_5_^-^ | 6.2 | C_4_H_5_O^-^ | 1.2 | C_15_H_19_O_3_^-^ | 2.5 | C_42_H_49_O_5_^-^ | 4.5 | C_25_H_20_O_5_^-^ | 11.0 |
|  |  |  |  |  |  | C_40_H_46_O_7_^-^ | 6.2 | C_4_H_10_^+^ | 1.1 |  |  | C_15_H_19_O_4_^-^ | 4.3 | C_9_H_17_^+^ | 9.3 |
|  |  |  |  |  |  | C_17_H_13_O_3_^-^ | 4.0 |  |  |  |  | C_15_H_20_O_4_^-^ | 3.6 | C_18_H_17_O_3_^-^ | 9.0 |
|  |  |  |  |  |  | C_46_H_57_O_6_^-^ | 3.4 |  |  |  |  | C_42_H_50_O_5_^-^ | 3.2 | C_45_H_37_O_2_^-^ | 8.3 |
|  |  |  |  |  |  | C_27_H_30_O_5_^-^ | 3.3 |  |  |  |  | C_23_H_33_O_4_^-^ | 3.2 | C_45_H_38_O_2_^-^ | 7.7 |
|  |  |  |  |  |  | C_27_H_29_O_5_^-^ | 3.3 |  |  |  |  | C_45_H_49_O_7_^-^ | 3.0 | C_15_H_23_O_3_^-^ | 5.0 |
|  |  |  |  |  |  | C_46_H_51_O_7_^-^ | 3.1 |  |  |  |  | C_27_H_23_O_4_^-^ | 2.5 | C_29_H_27_O_4_^-^ | 4.9 |
|  |  |  |  |  |  | C_43_H_30_O_10_^-^ | 2.9 |  |  |  |  | C_19_H_17_O_3_^-^ | 2.2 | C_43_H_30_O_8_^-^ | 4.9 |
|  |  |  |  |  |  | C_10_H_19_O^-^ | 2.4 |  |  |  |  | C_26_H_36_O_4_^-^ | 2.2 | C_7_H_7_O_3_^-^ | 4.8 |
|  |  |  |  |  |  | C_48_H_44_O_6_^-^ | 2.3 |  |  |  |  | C_44_H_46_O_8_^-^ | 2.1 | C_8_H_14_O_2_^-^ | 4.3 |
|  |  |  |  |  |  | C_27_H_37_O_5_^-^ | 2.2 |  |  |  |  |  |  | C_9_H_18_^+^ | 4.3 |


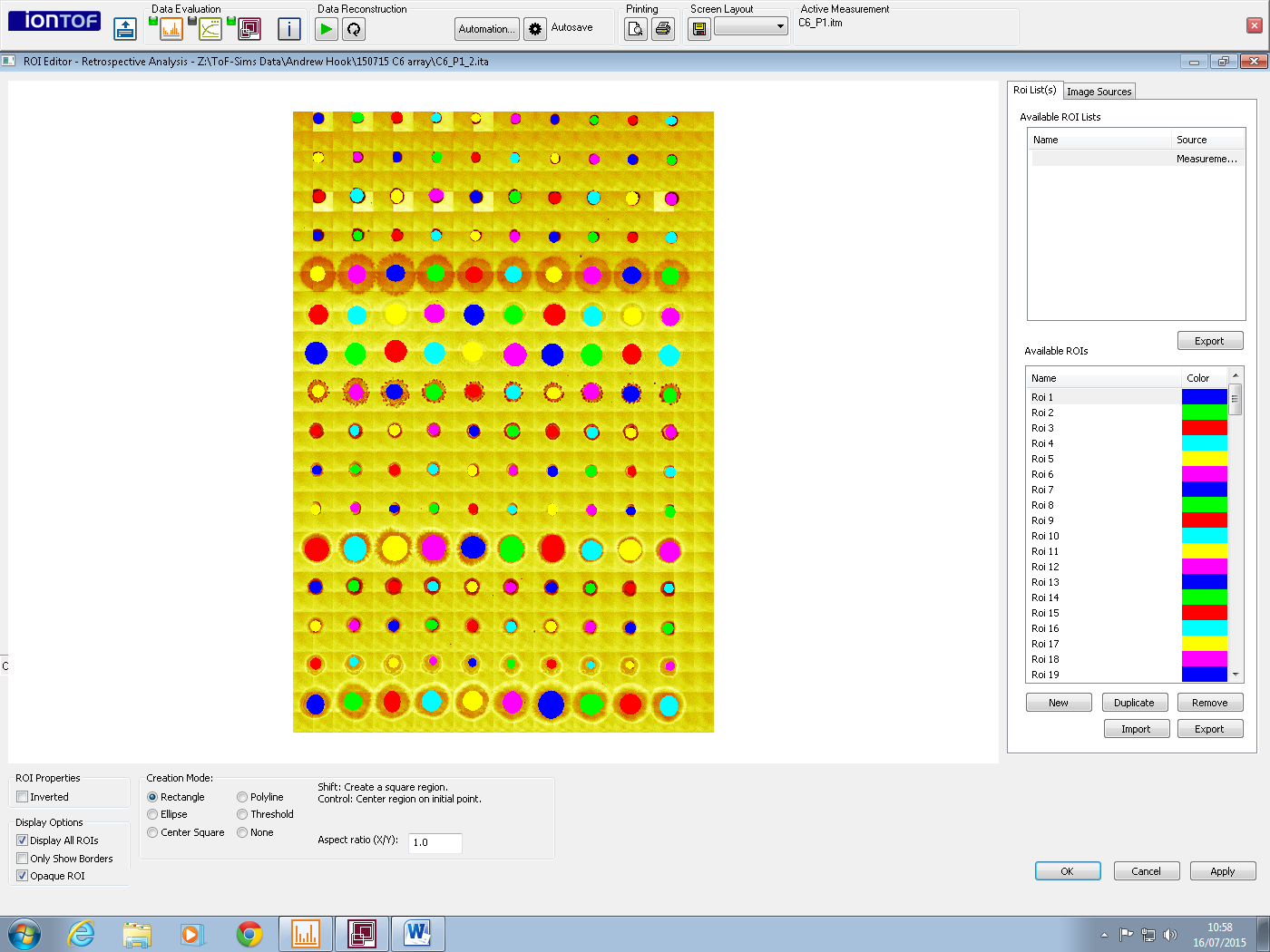


**Figure SI1.** ToF-SIMS total ion image of the polymer microarray with assigned regions of interest. Rows from top to bottom represents polymers A to P and left to right is 10 replicates of each material. The region of interest colour is non-indicative of polymer identity and is used only as a visual aid.

(A)

(B)

(C)

(D)

**Figure SI2.** ToF-SIMS spectra for polymers of monomers A-D. Mass range up to 200 shown. The spectra shown are the average taken from 10 separate samples. The left spectra were obtained for positive ions whilst the right spectra were obtained for negative ions.

(E)

(F)

(G)

(H)

**Figure SI2 (cont).** ToF-SIMS spectra for polymers of monomers E-H. Mass range up to 200 shown. The spectra shown are the average taken from 10 separate samples. The left spectra were obtained for positive ions whilst the right spectra were obtained for negative ions.

(I)

(J)

(K)

(L)

**Figure SI2 (cont).** ToF-SIMS spectra for polymers of monomers I-L. Mass range up to 200 shown. The spectra shown are the average taken from 10 separate samples. The left spectra were obtained for positive ions whilst the right spectra were obtained for negative ions.

(M)

(N)

(O)

(P)

**Figure SI2 (cont).** ToF-SIMS spectra for polymers of monomers M-P. Mass range up to 200 shown. The spectra shown are the average taken from 10 separate samples. The left spectra were obtained for positive ions whilst the right spectra were obtained for negative ions.

**Figure SI3.** Eigenvalue plot for the PCA. Using a scree test and considering the principal components that explained 95% of the variance, 9 principal components were selected for further analysis.


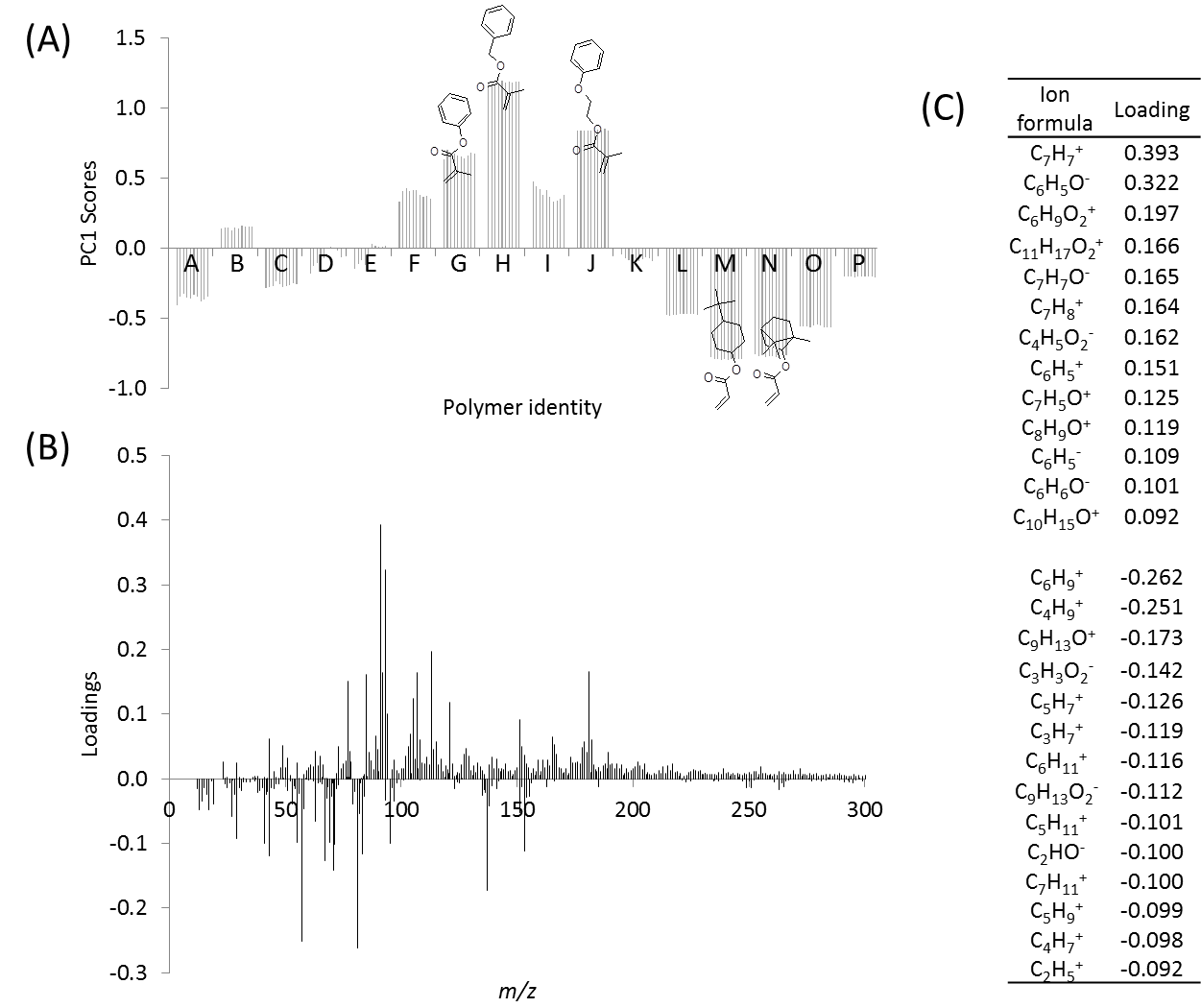


**Figure SI4.** Summary of PC 1 (captured 20% of the variance). (A) The scores plot for PC1 for each of the 16 materials. The structures for polymers with significant scores for PC1 are shown. (B) The loadings plot for PC1. (C) Table listing the ions for PC1 with the most positive or most negative loadings.


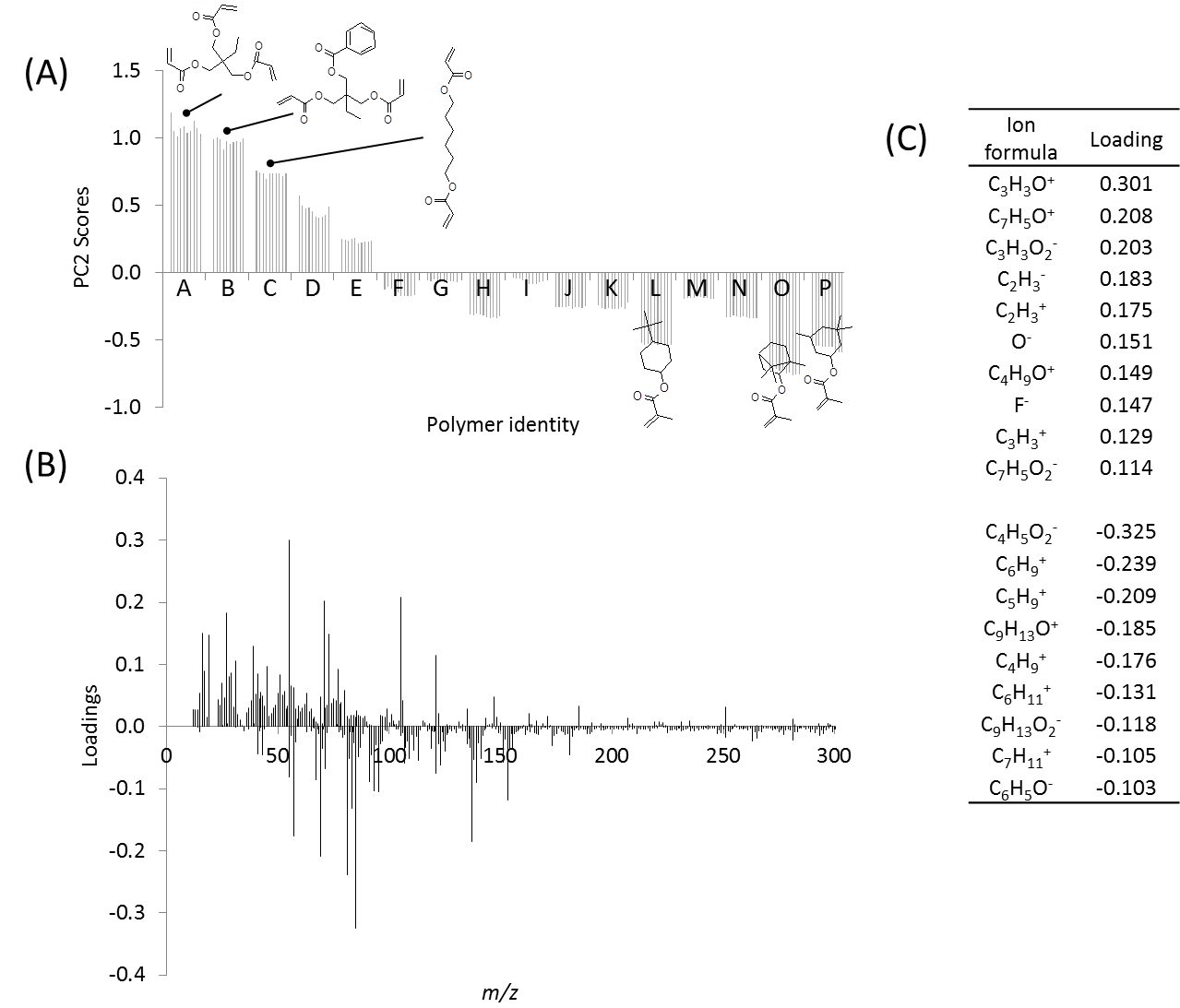


**Figure SI5.** Summary of PC2 (captured 19% of the variance). (A) The scores plot for PC2 for each of the 16 materials. The structures for polymers with significant scores for PC2 are shown. (B) The loadings plot for PC2. (C) Table listing the ions for PC2 with the most positive or most negative loadings.


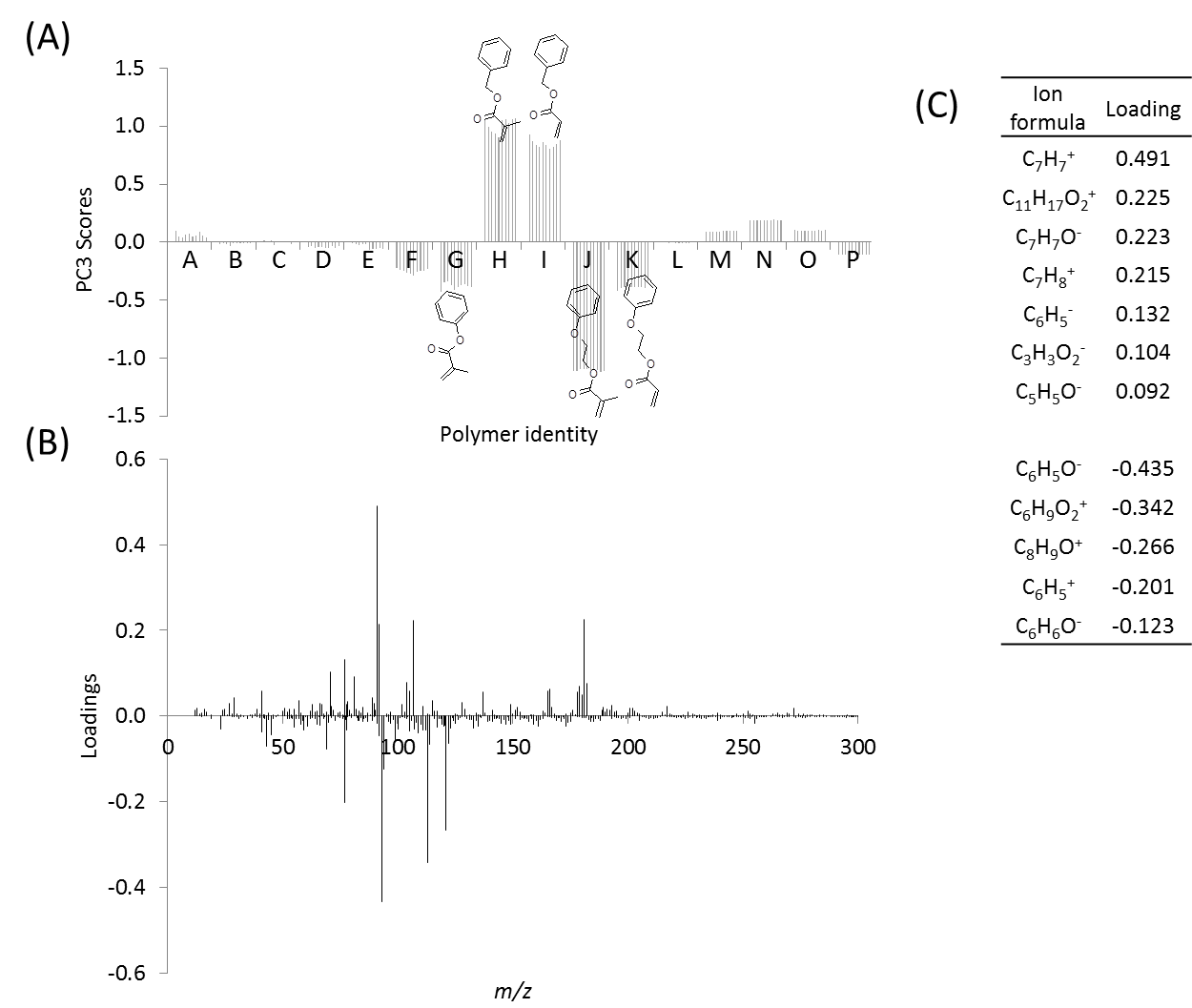


**Figure SI6.** Summary of PC3 (captured 14% of the variance). (A) The scores plot for PC3 for each of the 16 materials. The structures for polymers with significant scores for PC3 are shown. (B) The loadings plot for PC3. (C) Table listing the ions for PC3 with the most positive or most negative loadings.


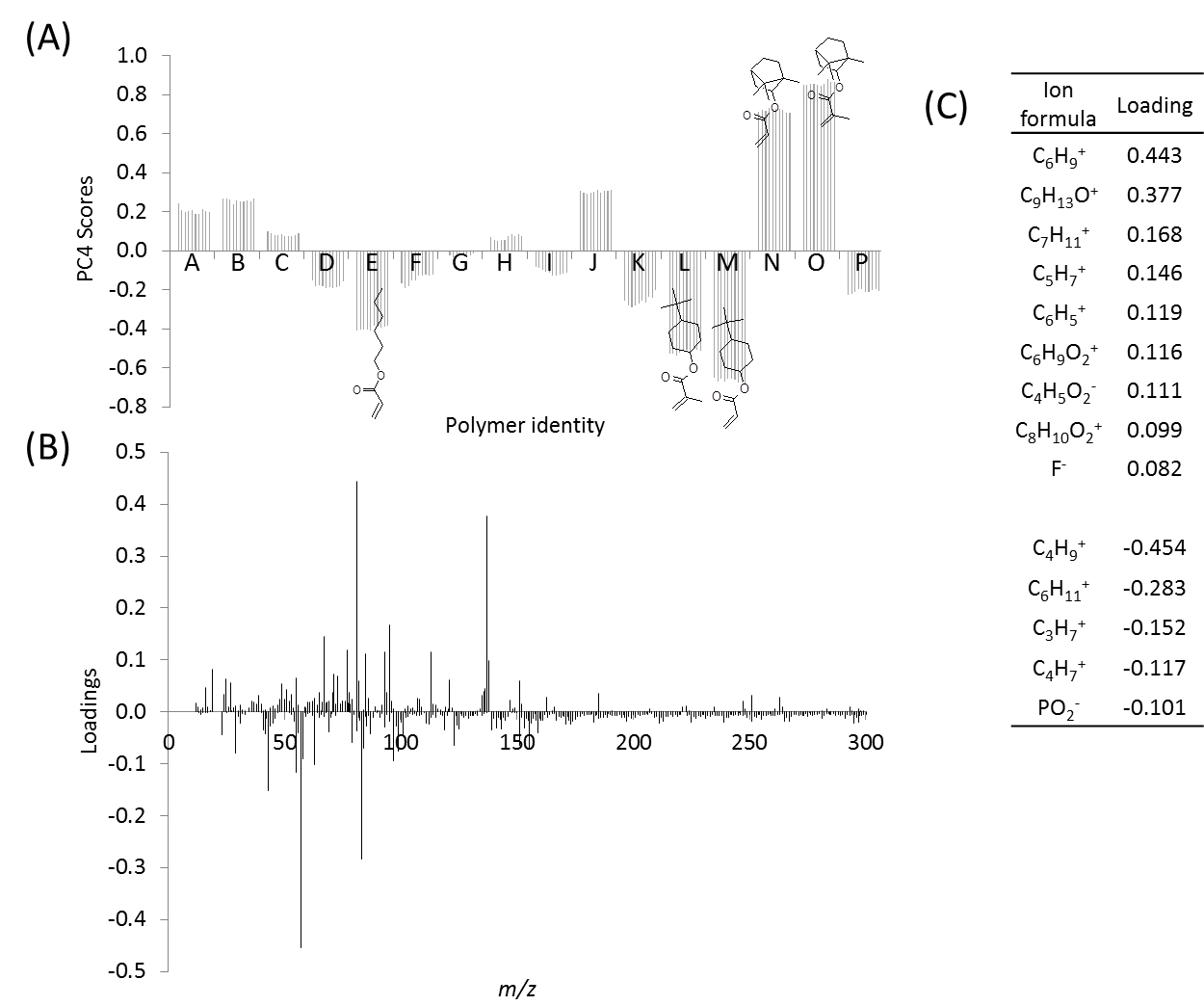


**Figure SI7.** Summary of PC4 (captured 10% of the variance). (A) The scores plot for PC4 for each of the 16 materials. The structures for polymers with significant scores for PC4 are shown. (B) The loadings plot for PC4. (C) Table listing the ions for PC4 with the most positive or most negative loadings.


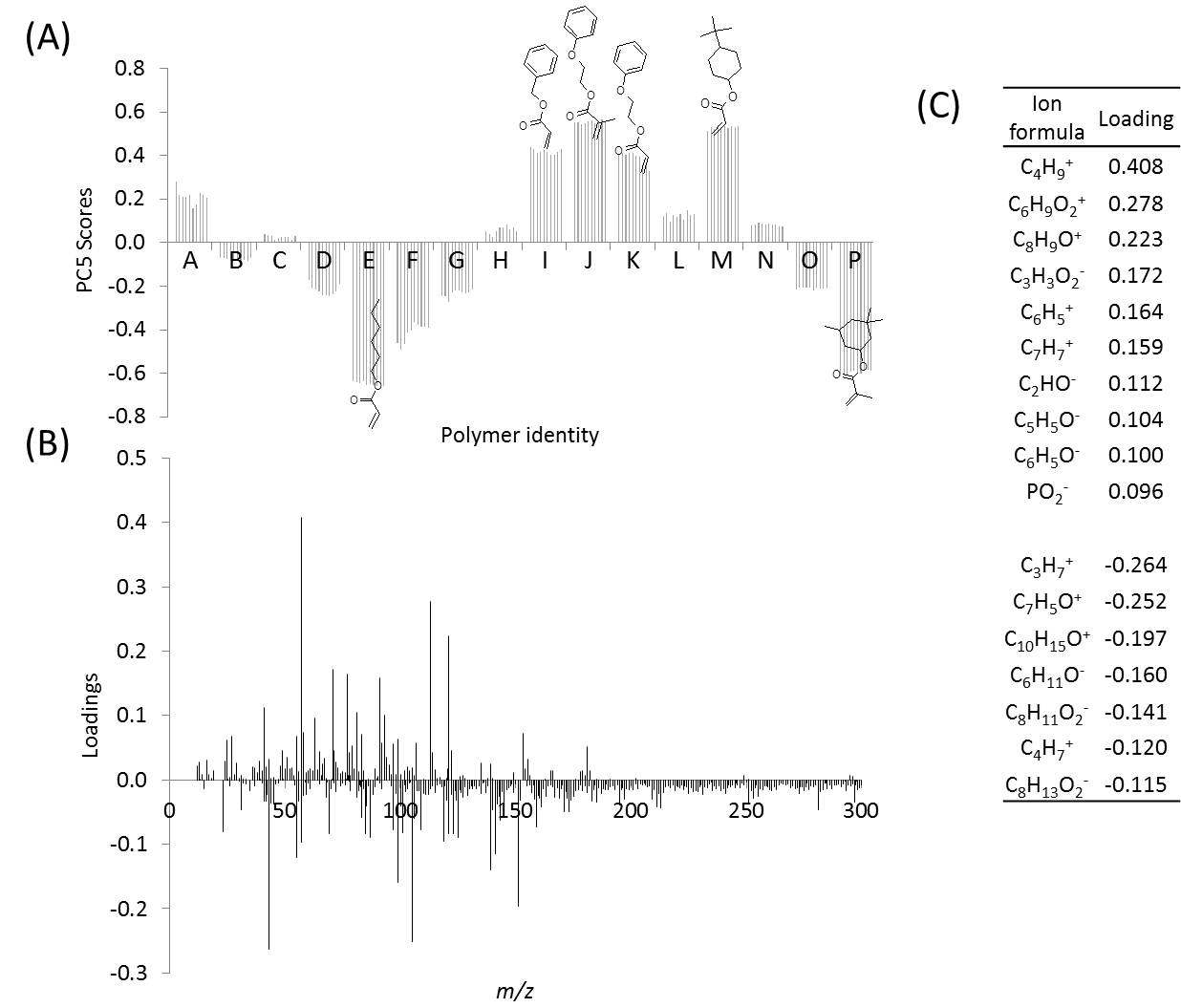


**Figure SI8.** Summary of PC5 (captured 9% of the variance). (A) The scores plot for PC5 for each of the 16 materials. The structures for polymers with significant scores for PC5 are shown. (B) The loadings plot for PC5. (C) Table listing the ions for PC5 with the most positive or most negative loadings.

**
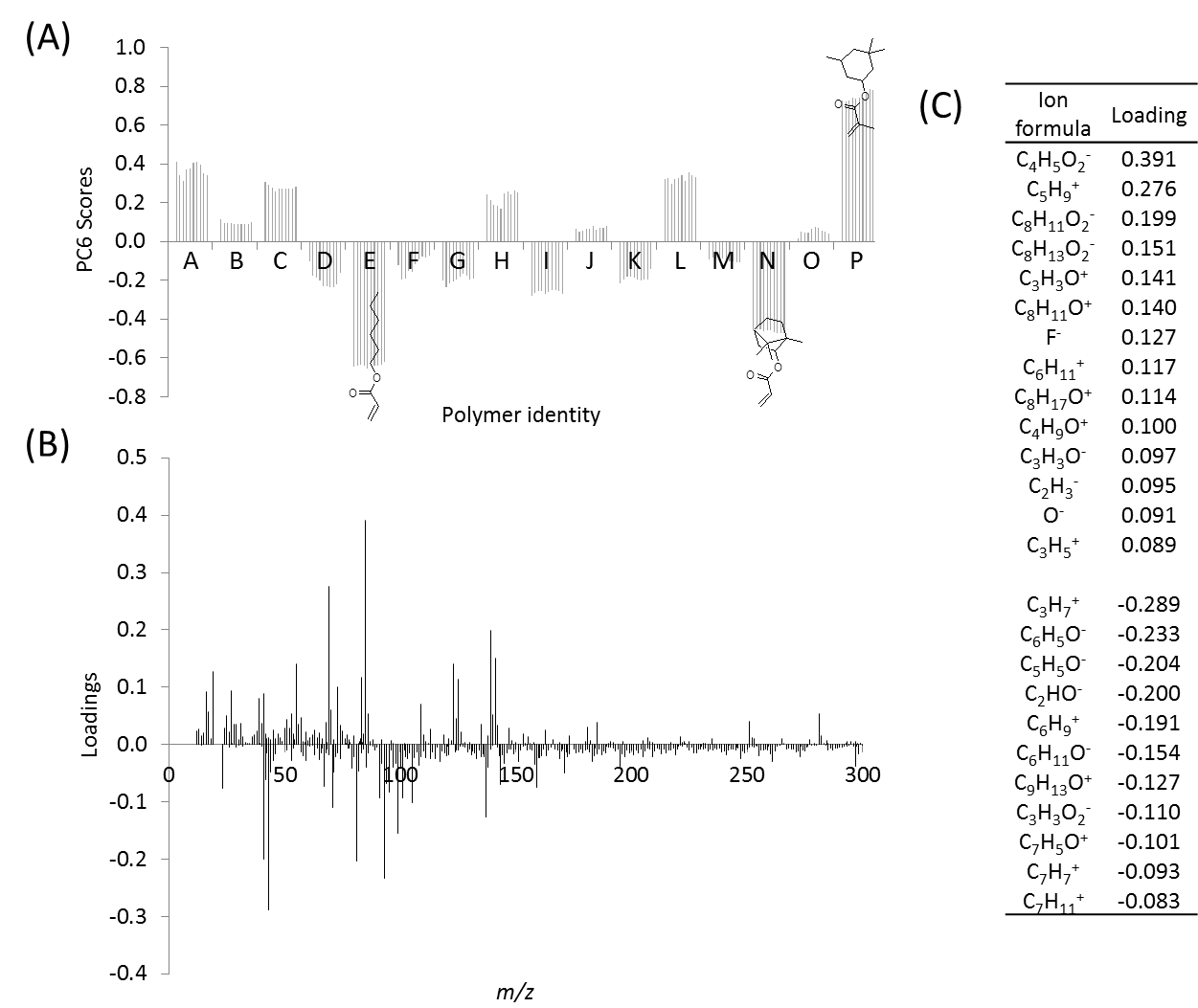
**

**Figure SI9.** Summary of PC6 (captured 7% of the variance). (A) The scores plot for PC6 for each of the 16 materials. The structures for polymers with significant scores for PC6 are shown. (B) The loadings plot for PC6. (C) Table listing the ions for PC6 with the most positive or most negative loadings.


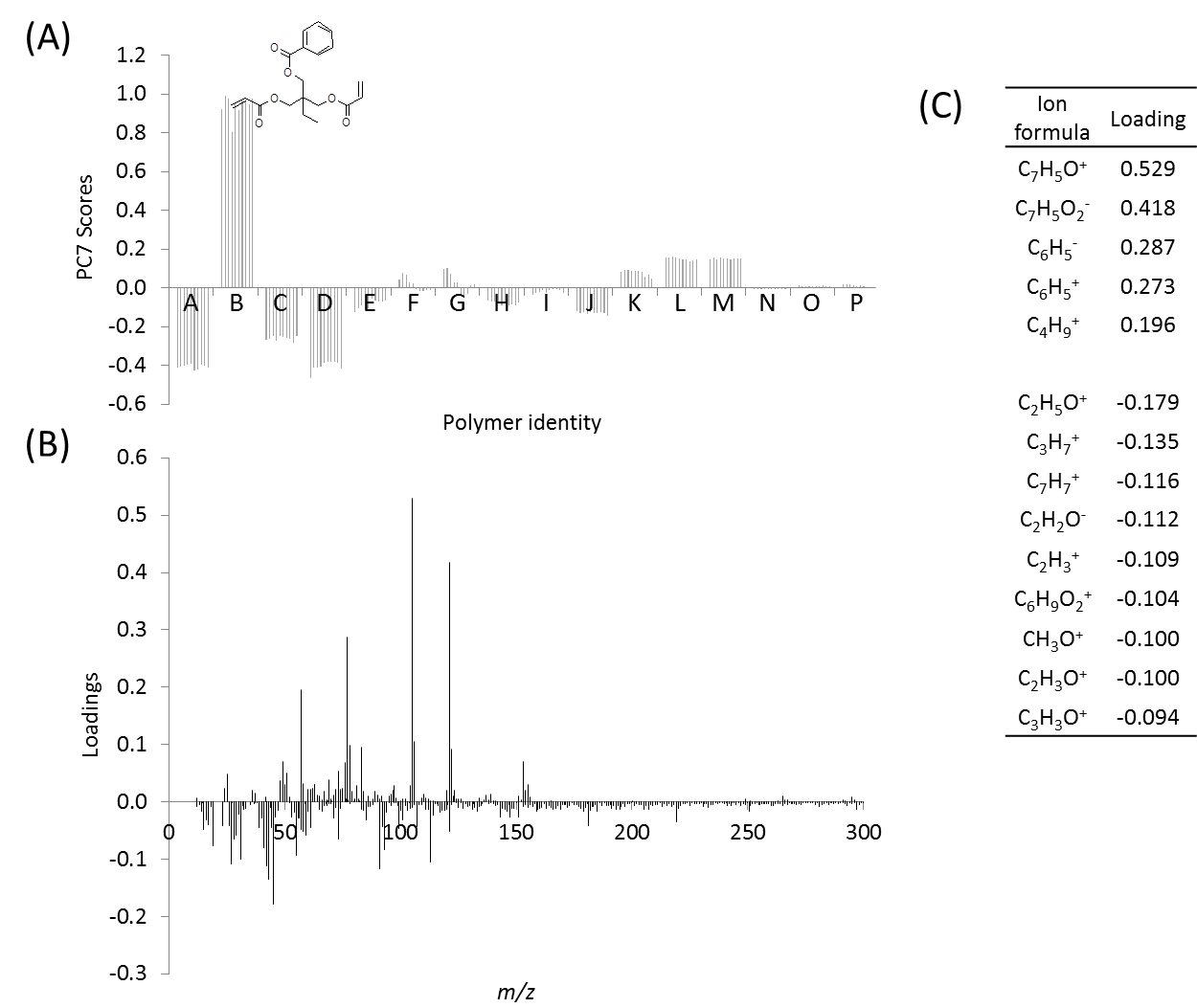


**Figure SI10.** Summary of PC7 (captured 6% of the variance). (A) The scores plot for PC7 for each of the 16 materials. The structures for polymers with significant scores for PC7 are shown. (B) The loadings plot for PC7. (C) Table listing the ions for PC7 with the most positive or most negative loadings.


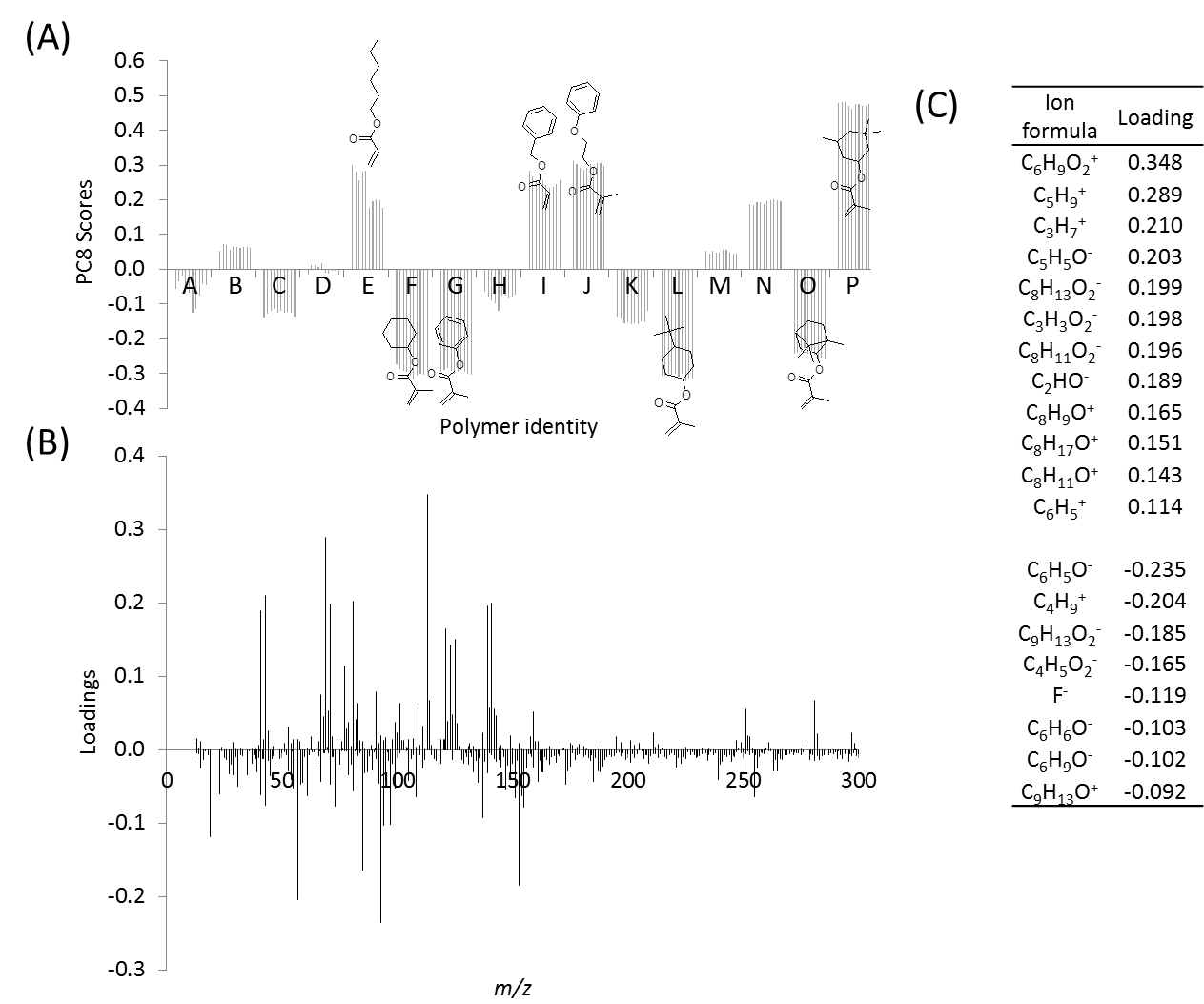


**Figure SI11.** Summary of PC8 (captured 4% of the variance). (A) The scores plot for PC8 for each of the 16 materials. The structures for polymers with significant scores for PC8 are shown. (B) The loadings plot for PC8. (C) Table listing the ions for PC8 with the most positive or most negative loadings.


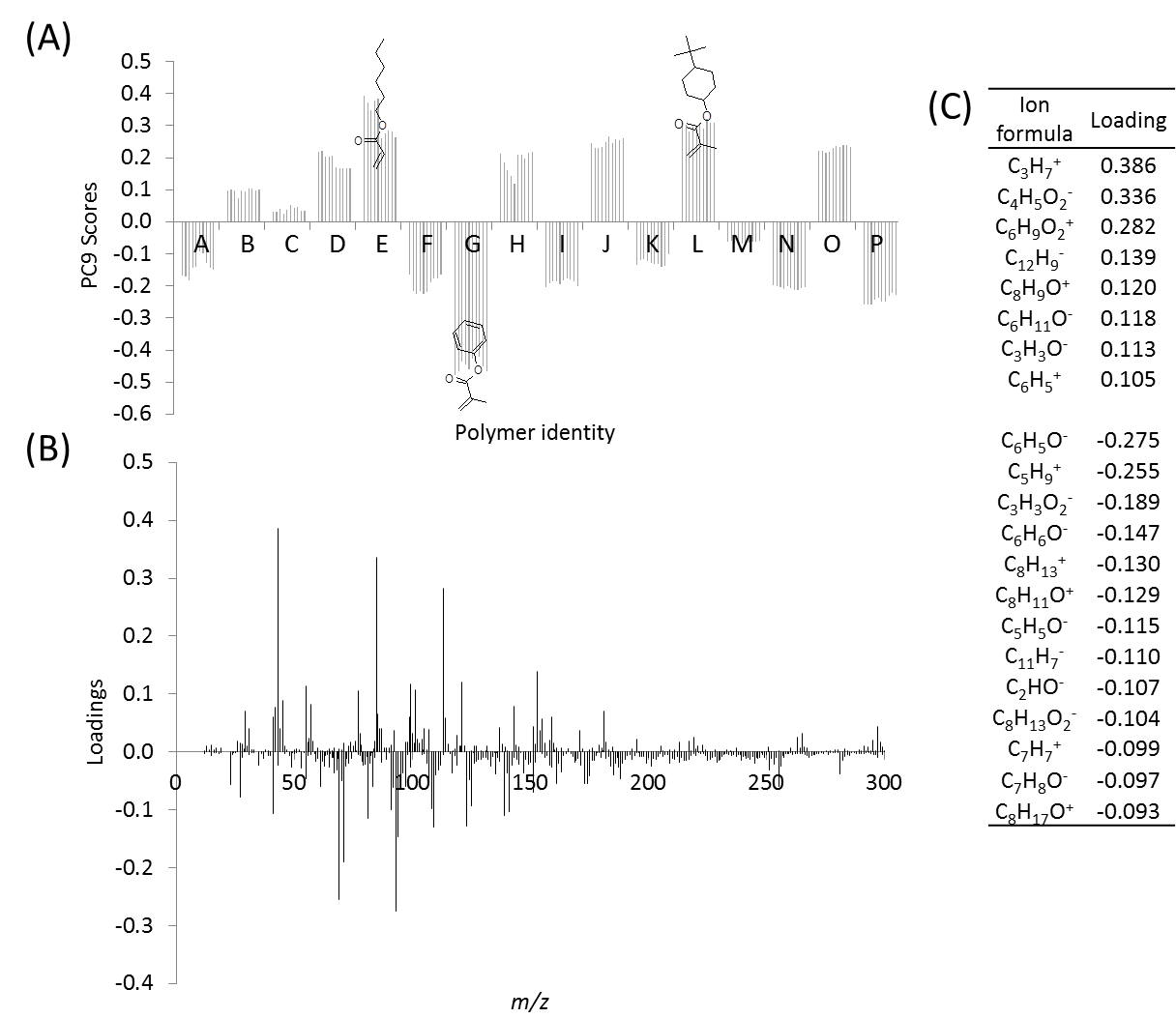


**Figure SI12.** Summary of PC9 (captured 3% of the variance). (A) The scores plot for PC9 for each of the 16 materials. The structures for polymers with significant scores for PC9 are shown. (B) The loadings plot for PC9. (C) Table listing the ions for PC9 with the most positive or most negative loadings.

**
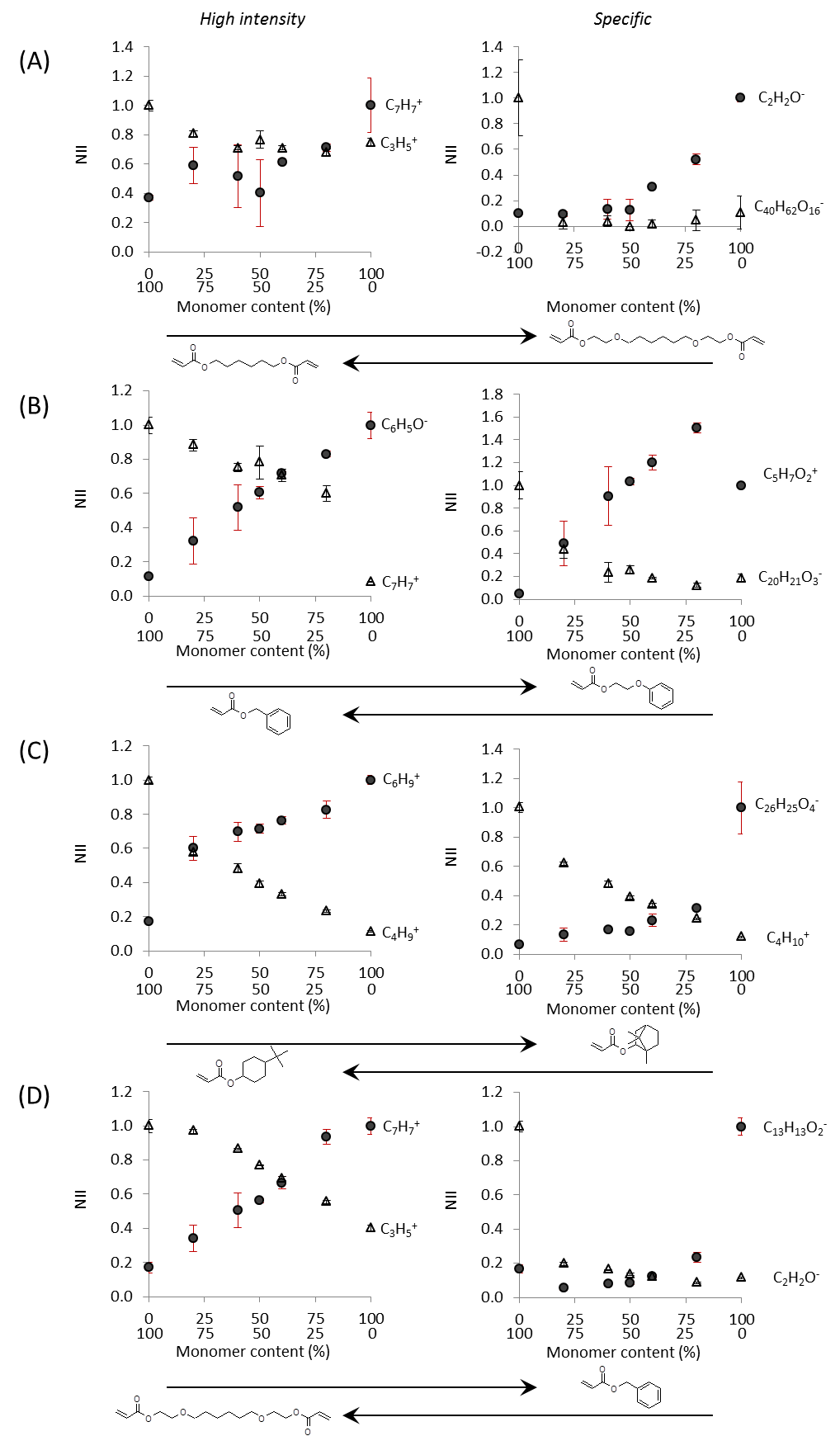
**

**Figure SI13. (**Continued next page)

**
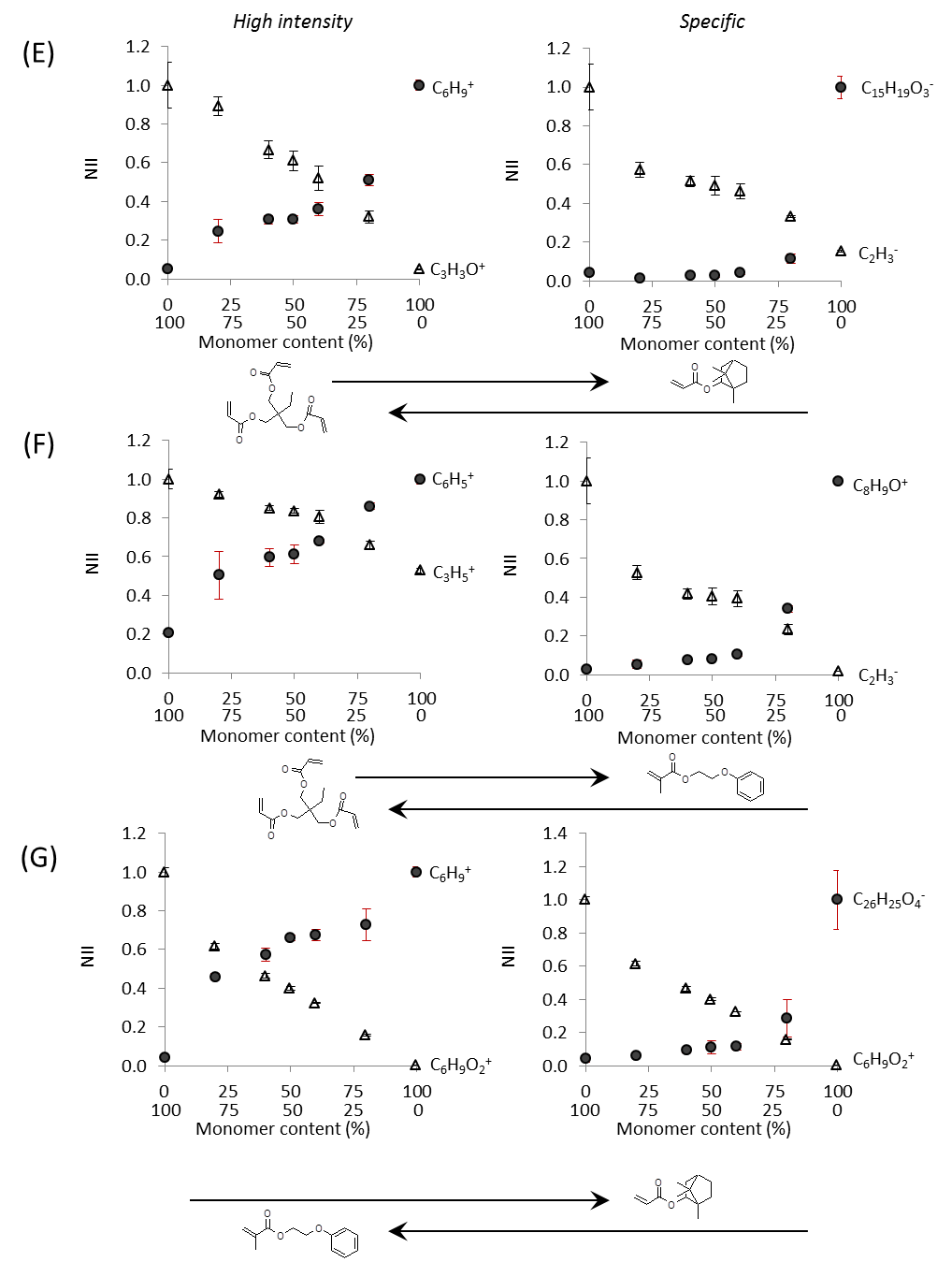
**

**Figure SI13.** Normalised ion intensity for (left) high intensity ions and (right) specific ions chosen from the homopolymers for the copolymer pairs of (A) *c* and *d*, (B) *i* and *k*, (C) *m* and *n*, (D) *d* and *i*, (E) *a* and *n*, (F) *a* and *j*, and (G) *j* and *n* for varied monomer content. Ion plotted indicated on each graph. Ion intensity first normalised to the total counts for ions identified from a peak search of the homopolymer library, and then normalised to the intensity measured for the homopolymer associated with the ion. Error bars equal ± one standard deviation unit, n = 10 for homopolymers, n = 3 for copolymers. Error bars associated with ‘●’ shown as red.

(A)

(B)

(C)

(D)

**Figure SI14. (**Continued next page)

(E)

(F)

(G)

**Figure SI14.** Scores plot of PC1 and 2 (left) and PC3 and 4 (right) for copolymers of monomers (A) *c* and *d*, (B) *i* and *k*, (C) *m* and *n*, (D) *d* and *i*, (E) *a* and *n*, (F) *a* and *j*, and (G) *j* and *n* at ratios of 1:0 (**×**), 4:1 (⯁), 3:2 (⯁), 1:1 (⯁), 2:3 (⯁), 1:4 (**◇**), and 0:1 (**△**).
